# Potential of pre-diagnostic metabolomics for colorectal cancer risk assessment or early detection

**DOI:** 10.1038/s41698-024-00732-5

**Published:** 2024-10-27

**Authors:** Teresa Seum, Clara Frick, Rafael Cardoso, Megha Bhardwaj, Michael Hoffmeister, Hermann Brenner

**Affiliations:** 1 Division of Clinical Epidemiology and Aging Research, German Cancer Research Center (DKFZ), Im Neuenheimer Feld 581, 69120 Heidelberg, Germany; 2 Medical Faculty Heidelberg, Heidelberg University, Im Neuenheimer Feld 672, 69120 Heidelberg, Germany; 3 German Cancer Consortium (DKTK), German Cancer Research Center (DKFZ), Im Neuenheimer Feld 280, 69120 Heidelberg, Germany

**Keywords:** Predictive markers, Risk factors, Cancer screening

## Abstract

This systematic review investigates the efficacy of metabolite biomarkers for risk assessment or early detection of colorectal cancer (CRC) and its precursors, focusing on pre-diagnostic biospecimens. Searches in PubMed, Web of Science, and SCOPUS through December 2023 identified relevant prospective studies. Relevant data were extracted, and the risk of bias was assessed with the QUADAS-2 tool. Among the 26 studies included, significant heterogeneity existed for case numbers, metabolite identification, and validation approaches. Thirteen studies evaluated individual metabolites, mainly lipids, while eleven studies derived metabolite panels, and two studies did both. Nine panels were internally validated, resulting in an area under the curve (AUC) ranging from 0.69 to 0.95 for CRC precursors and 0.72 to 1.0 for CRC. External validation was limited to one panel (AUC = 0.72). Metabolite panels and lipid-based biomarkers show promise for CRC risk assessment and early detection but require standardization and extensive validation for clinical use.

## Introduction

Colorectal cancer (CRC) is the second leading cause of cancer-related death worldwide, with an estimated 1.9 million incident cases and 904,000 deaths in 2022^1^. CRC often progresses slowly from precancerous to malignant neoplastic lesions, offering opportunities for prevention and enhanced prognosis by early detection and removal of precancerous lesions or detection and treatment of cancer at an earlier stage^2^. Various screening modalities have been developed for early detection of CRC and its precursors, including colonoscopy or fecal blood tests^3^. While colonoscopy is considered the gold standard for early detection of CRC and its precursors due to its high sensitivity and specificity, it is invasive, carries a risk of complications, and has low adherence^4^. Fecal blood tests are noninvasive but have limited sensitivity for early-stage CRC and precursors of CRC and are recommended every one to three years^5–7^. Despite the availability of these screening modalities, the development of further noninvasive methods with enhanced acceptability, accessibility, and performance would be highly desirable.

In recent years, metabolomics has emerged as a promising approach for cancer screening, including CRC. Metabolomics involves the systematic study of small molecule metabolites in biological fluids, cells, and tissues, and research on its potential application in the field of cancer biomarker discovery is rapidly expanding^8,9^. Previous studies using metabolomics have shown promise in differentiating individuals with and without CRC^10^. However, most studies have assessed metabolomics after CRC diagnosis and were carried out in clinical settings, which may limit their relevance for general population screening since it may reflect secondary changes in the metabolome after the onset of symptoms and diagnosis of CRC^11^. Studies conducted to identify and validate metabolite biomarkers for CRC risk based on pre-diagnostic biospecimens may help identify more effective and less invasive screening methods for CRC. Therefore, the aim of this systematic review is to evaluate the existing evidence on metabolite biomarkers for CRC or its precursors, which were identified in pre-diagnostic samples, such as in prospective cohorts or in a screening setting.

## Results

### Literature search result

**Fig. 1:**
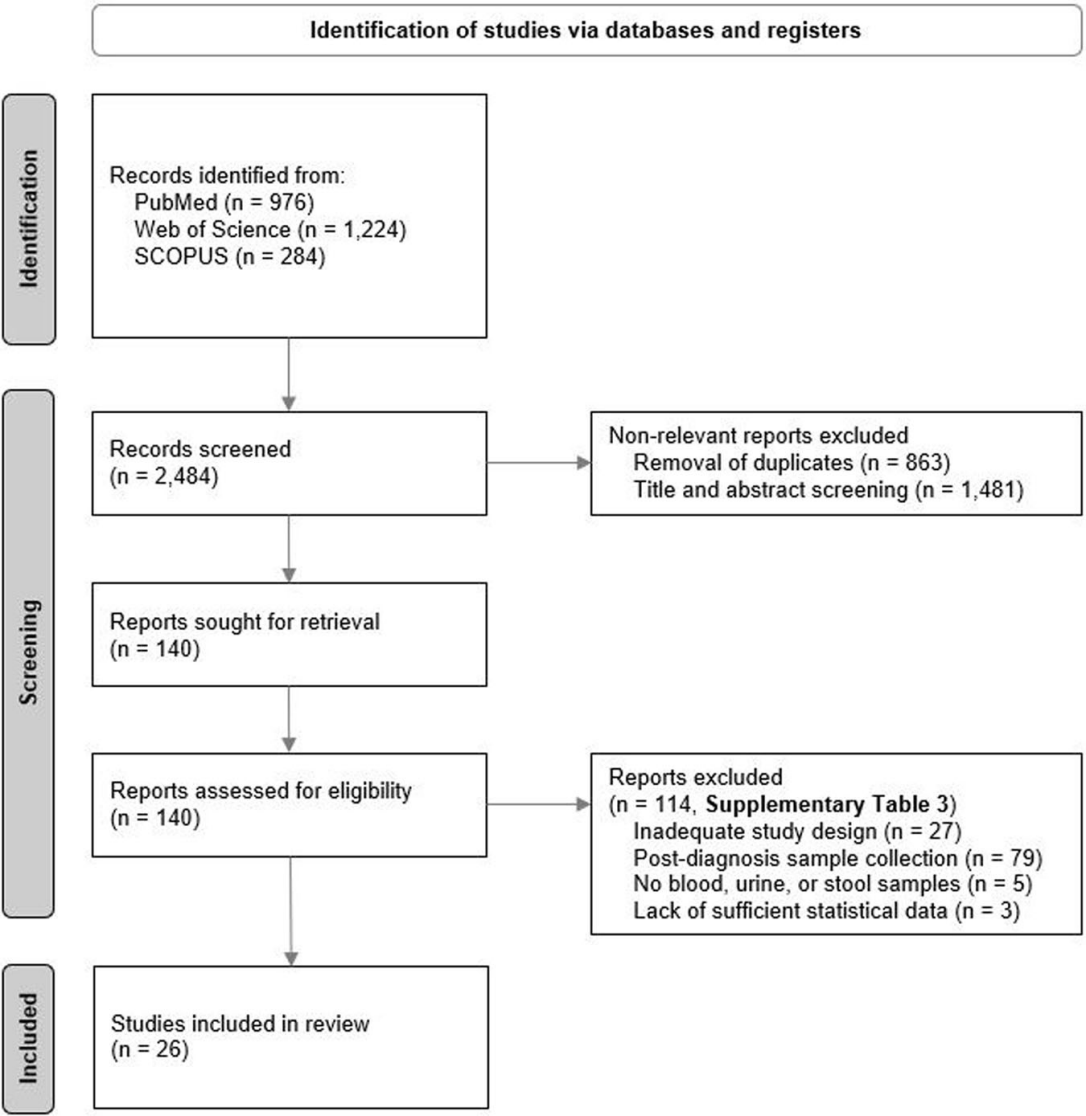
Preferred reporting items for systematic reviews and meta-analyses (PRISMA) flow diagram.

The comprehensive literature search across the specified databases using the predefined search terms resulted in a total of 2,484 records. A detailed overview of the selection process is depicted in the PRISMA flow diagram shown in Fig. 1. After applying the eligibility criteria, 140 articles were chosen for an in-depth full-text review. Among these articles, 27 were excluded due to inadequate study design, 79 were excluded as the individuals were already diagnosed with CRC or a precursor at the time of biospecimen collection, five were excluded due to studied biospecimens others blood, urine, or stool, and three were excluded due to insufficient statistical data. The references of the studies excluded are listed in Supplementary Table 3. In the end, 26 studies focusing on the predictive performance of metabolite biomarkers, published up to December 30, 2023, were incorporated into this systematic review.

### Study characteristics

Details on study characteristics are summarized in Table 1. The investigated outcomes comprised CRC in a total of 14 studies^12–25^, colon cancer in two studies^26,27^, adenomas in two studies^28,29^, polyps in four studies^30–33^, a combination of adenomas and polyps in two studies^34,35^, and a combination of adenomas and CRC in two studies^36,37^. The studies reviewed focused on individual metabolites (13 studies^12–15,17–19,24,26,27,34,35,37^) and metabolite panels (eleven studies^20,22,23,25,28–33,36^) for differentiating CRC or its precursors from controls. The studies reporting on individual metabolites utilized a variety of designs: two were screening trials^35,37^, six were nested case-control studies^12,13,15,18,19,26^, three were prospective cohort studies^17,24,34^, and two articles reported on both a cohort and a screening study^14,27^. Studies reporting on metabolite panels included nine screening studies^22,23,25,29–33,36^, one prospective cohort^28^, and one nested case-control study^20^. Additionally, two nested case-control studies investigated both individual metabolites and metabolite panels^16,21^.

**Table 1:** Details of included studies reporting on the prediction of the presence or occurrence of CRC using metabolomics

First author, Year ^ref.^	Study type country	Study group	Time to diagnosis^a^ (mean)	Population			Validation approach	
				*N*	Age (mean, SD)	Female (%)	IV	EV
**Individual Metabolites**								
Cai (2006) ^12^	Nested case-control China	CRC, CC, RC	30 m	150	60.3 (8.3)	100	-	-
		Cn	-	150	60.1 (8.5)	100		
Cross (2014) ^13^	Nested case-control USA	CRC	7.8 y ^med^	254	64.3 (5.1)	44.1	-	-
		Cn	-	254	64.3 (5.1)	43.7		
Kühn (2016) ^15^	Nested case-control Germany	CRC	6.57 y ^med^	163	55.8 (6.4)	37.4	-	-
		Cn ♀	-	348	52.3 (7.1)	100		
		Cn ♂	-	426	49.1 (8.5)	0		
Myte (2017) ^18^	Nested case-control Sweden	CRC	8.2 (4.7–11.9) y ^med, IQR^	613	59.3 (40.1–67.8) ^med, IQR^	59	Boot- strapping	-
		Cn	-	1190	59.7 (40.0–67.8) ^med, IQR^	59		
Pickens (2017) ^35^	Screening USA	A	N/A	37	58 (53–60) ^med, IQR^	0	-	-
		HPP	-	20	58 (53–60) ^med, IQR^	0		
		Cn	-	69	57 (53–61) ^med, IQR^	0		
Geijsen (2019) ^14^	Prospective cohort/screening Germany and Austria	CRC	N/R	180	66.0 (58.0–73.0) ^med, IQR^	36.7	-	Yes
		Cn	-	153	51.0 (42.0–63.0) ^med, IQR^	61.4		
		CRC *(EV)*	N/R	88	70.0 (60.0–76.0) ^med, IQR^	31.8		
		Cn *(EV)*	-	200	64.0 (57.0–74.0) ^med, IQR^	35.0		
Kühn (2020) ^26^	Nested case-control Europe	CC	N/R	569	57.5 (36.7–74.3) ^med, range^	62.6	-	-
		Cn	-	569	57.5 (36.7–74.3) ^med, range^	62.6		
McCullough (2021) ^17^	Prospective cohort USA	CRC	N/R	517	70.2 (5.5)	44.3	-	-
		Cn	-	517	70.2 (5.5)	44.3		
Papadimitriou (2021) ^27^	Prospective cohort/screening Germany and Austria	CC (ColoCare)	N/R	110	65 (13)	39	-	Yes
		Cn (ColoCare)	-	153	51 (15)	61		
		CC (CORSA)	N/R	46	69 (14)	28		
		Cn (CORSA)	-	390	63 (13)	35		
		CC (EPIC)	6.6 (3.5) y	456	56 (7.8)	N/R		
		Cn (EPIC)	-	456	56 (7.7)	N/R		
Tevini (2022) ^37^	Screening Austria	CRC	N/A	18	67 (12)	38.9	Split sampling	-
		AA	-	28	60 (10)	50		
		Cn	-	36	53 (8)	50		
		CRC *(IV)*	N/A	48	69 (10)	35.4		
		Cn for CRC *(IV)*	-	29	68 (7)	89.7		
		AA *(IV)*	-	48	66 (10)	45.83		
		Cn for AA *(IV)*	-	28	66 (5)	0		
Hang (2022) ^34^	Prospective cohort USA	A	N/A	586	53.6 (7.8)	100		
		Cn for A	-	1141	53.8 (7.8)	100		
		SP	N/A	509	52.9 (7.5)	100		
		Cn for SP	-	993	53.1 (7.5)	100		
Pham (2022) ^19^	Nested case-control Europe	CRC	4.8 (2.7) y	1,293	58.1 (7.0)	52.7	-	-
		Cn	-	1,293	58.1 (7.0)	52.7		
Vidman (2023) ^24^	Nested case-control Sweden	CRC	10.3 y	902	56.2 (7.4)	48.8	Cross-validation	-
		Cn	-	902	56.2 (7.4)	48.8		
**Metabolite panels**								
Eisner (2013) ^32^	Screening Canada	P	N/A	355	58.9 (8.2)	44.79	Cross-validation	-
		Cn	-	633	56.2 (8.1)	57.5		
Wang (2014) ^33^	Screening Canada	AP	N/A	422	55.7 (0.4)	41	Split sampling	-
		Cn	-	162	59.1 (0.6)	57		
		AP *(IV)*	N/A	211	56.1 (0.6)	38		
		Cn *(IV)*	-	81	60.4 (0.8)	58		
Amiot (2015) ^36^	Screening France	AA/CRC	N/A	33	59.4 ( ± 6.9) ^med, IQR^	24	Cross-validation	-
		Cn		22	52.0 ( ± 12.0) ^med, IQR^	32		
Farshidfar (2016) ^28^	Prospective cohort Canada	A	N/R	31	59.5 (5.9)	32	Cross-validation	-
		Cn	-	31	60.5 (6.7)	28		
Deng (2017a) ^30^	Screening Canada	AP	N/A	155	59.9 (7.4)	38.7	Split sampling	-
		Cn	-	530	56.1 (8.2)	58.1		
Deng (2017b) ^31^	Screening China	AP *(EV)*	N/A	345	65.1 (6.6)	43	-	Yes
		Cn *(EV)*	-	316	61.8 (7.4)	74		
Troisi (2022) ^23^	Screening Italy	CRC	N/A	100	66.2 (11.3)	36	Split sampling	-
		BCT	N/A	50	62.8 (7.1)	41		
		Cn	-	50	61.6 (7.0)	44		
Rothwell (2022) ^20^	Nested case-control Europe	CRC	7.7 (4.4) y	1,608	56.9 (7.5)	45.4	-	-
		Cn	-	1,608	56.8 (7.5)	45.4		
Telleria (2022) ^22^	Screening Spain	CRC	N/A	40	73.0 (11.3)	50	Split sampling	-
		AA	-	40	70.4 (10.8)	50		
		Cn	-	40	66.2 (14.1)	50		
Liu (2023) ^29^	Screening USA	CTC	N/A	23	N/R	N/R	Cross-validation	-
		Cn	-	20	N/R	50		
Xie (2023) ^25^	Screening China	CRC	N/A	35	57 (37–81) ^med, range^	45.7	-	-
		Cn	-	30	45 (23‑67) ^med, range^	60.0		
**Individual metabolites & metabolite panels**								
Shu (2018) ^21^	Nested case-control China	CRC ♀	N/R	122	56.9 (8.4)	100	-	-
		Cn ♀	-	122	57.0 (8.4)	100		
		CRC ♂	N/R	123	56.2 (6.8)	0		
		Cn ♂	-	123	56.5 (6.6)	0		
Loftfield (2022) ^16^	Nested case-control USA	CRC ♀	10 y	233	64.2 (5.3)	100	-	-
		Cn ♀	-	233	64.1 (5.3)	100		
		CRC ♂	10 y	262	64.0 (5.0)	0		
		Cn ♂	-	262	64.0 (5.1)	0		

*(A)A* (advanced) adenoma, *AP* colonic adenomatous polyps, *BCT* benign colorectal tumor, *Cn* controls, *CC* colon cancer, *CTC* colonic tumor carriers, *CRC* colorectal cancer, *SP* serrated polyps, *HPP* hyperplastic polyps, *P* polyps, *RC* rectal cancer, *SD* standard deviation, ^*med*^ median, ^*IQR*^ interquartile range, *y* years, *m* months, *N/A* not applicable, *N/R* not reported, *IV* internal validation, *EV* external validation, ♀ female, ♂ male.

^a^only applicable for cohort studies and for the outcome CRC/CC.

Besides four studies from China^12,21,25,31^, all studies were conducted in predominately white populations. Six studies were conducted in the United States^13,16,17,29,34,35^, four in Canada^28,30,32,33^, and 12 in European countries—five spanned several European countries^14,19,20,26,27^, and seven took place in single European countries, including Italy^23^, Sweden^18,24^, Spain^22^, France^36^, Austria^37^, and Germany^15^.

Two studies exclusively included females^12,34^ while one study focused solely on males^35^. The male to female proportion among cases varied across studies, with three reporting more female cases^18,19,26^, 18 reporting more male cases^13–17,20,21,23–25,27,28,30–33,36,37^, one reporting an equal proportion of males and females^22^, and one not specifying the sex distribution of participants^29^.

The number of CRC cases varied widely, ranging from 18 cases^37^ to 1608 cases^20^. For adenoma cases, the range was from 23 cases^29^ to 586 cases^34^, while for polyps, the range extended from of 20 cases^35^ to 355 cases^32^. Matching of cases and controls was employed in 13 studies, incorporating criteria such as age, sex, ethnicity, year of randomization, season of blood draw, recruitment time point, time period of endoscopy, fasting status, study cohort, smoking status, and menopausal status^12–14,16–21,24,26–28,34^.

The biospecimens utilized in the investigations included mainly blood (serum in seven studies^13,16,19,23,28,29,37^, plasma in ten studies^14,15,17,18,21,24,26,27,34,35^, combination of serum and plasma in one study^20^), urine in five studies^12,30–33^, and stool in three studies^22,25,36^. Technologies used for metabolomics analyses were mainly liquid chromatography–mass spectrometry (LC–MS), which was used by 9 studies^12,16,17,22,24,26,27,30,34^, and other mass-spectrometry (e.g., flow injection analysis–tandem mass spectrometry, isobaric labeling mass spectrometry)^14,23,25,28,29^, or a combination of mass spectrometry with a different technology^13,15,18,20,21,37^. Other techniques used were gas chromatography (GC)^35^, nuclear magnetic resonance (NMR)^31–33,36^, and ELISA assay^19^.

### Validation techniques to address overoptimism

Validation efforts to mitigate overoptimism in model predictions were reported by 14 out of the 26 studies, with methodologies outlined in Table 1. These studies employed various validation techniques to enhance the reliability of their findings. Split-sampling method was utilized in five different studies^22,23,30,33,37^. More advanced techniques, including different types of cross-validation^24,28,29,32,36^ and bootstrapping^18^ were used by six studies. External validation was performed by three studies, two evaluated individual metabolites^14,27^, and one focused on a metabolite panel^31^.

### Performance of individual metabolites and metabolite panels

**Fig. 2:**
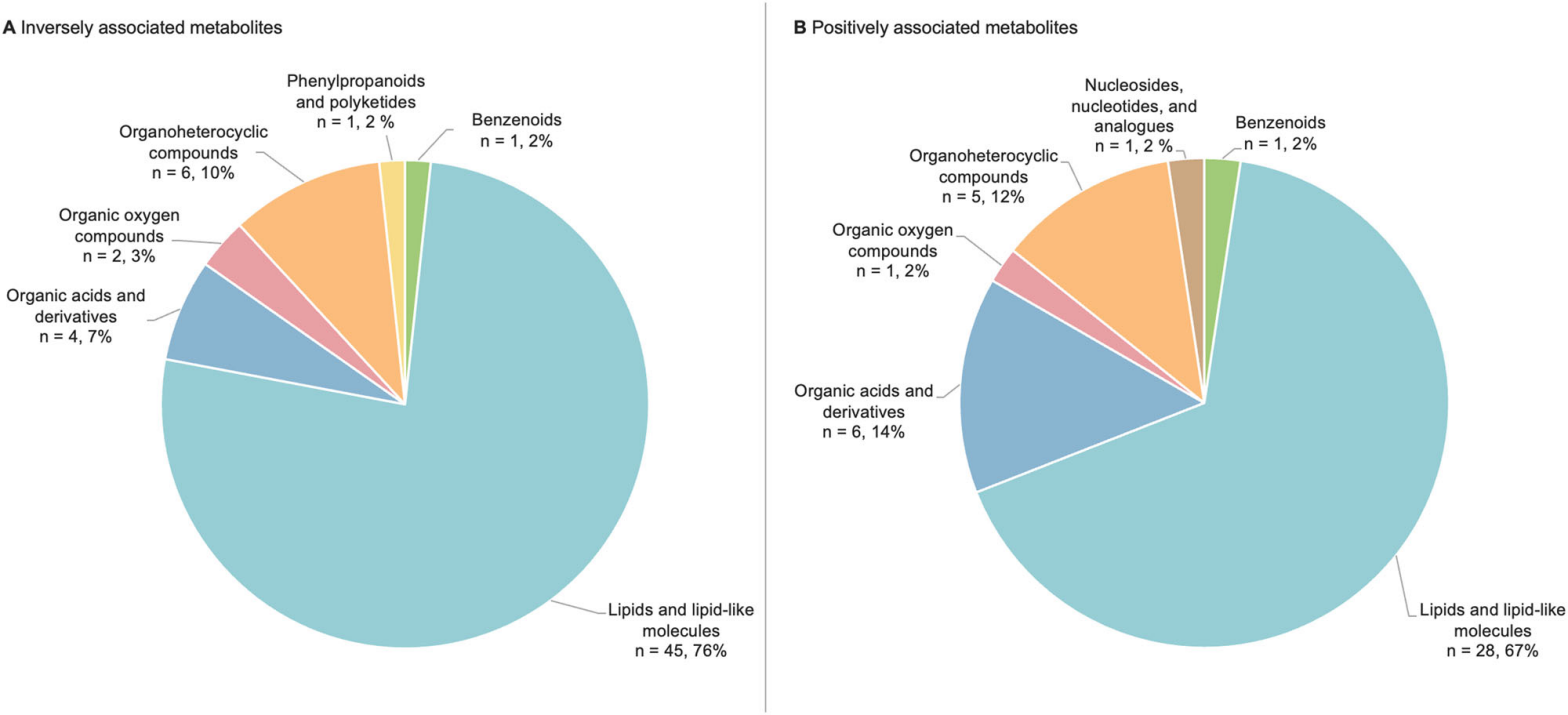
Associations between individual metabolites and colorectal cancer risk, categorized by direction of association. **A** Inversely associated metabolites with colorectal cancer risk. **B** Positively associated metabolites with colorectal cancer risk. Note: metabolites are grouped by Super Class from the Human Metabolome Database. Metabolites reportedas ratios are excluded.

Potential metabolite biomarkers for prediction or diagnosis of CRC were found in different biospecimen types (blood, urine, stool) and varied in their biochemical classes. Half of the included studies reported on the performance of individual metabolites (13 out of 26), eleven studies reported on a panel of metabolites, and two reported on the performance of individual metabolites as well as the performance of a panel. Table 2 shows the individual metabolite biomarkers for CRC and their precursors, identified by different analytical approaches. Six studies used an untargeted approach to discover the metabolites^13,14,17,21,24,34^, while the other nine studies used a targeted approach to measure predefined metabolites^12,15,16,18,19,26,27,35,37^. Three of the 15 studies reporting on individual metabolites did not find any significant associations between the metabolites studied and CRC^13,15,19^. The remaining twelve studies reported significant associations for a total of 101 metabolites (Fig. 2). Among the 59 metabolites inversely associated with CRC, two-thirds (*n* = 45, 76%) were lipids or lipid-like molecules. Organoheterocyclic compounds and organic acids and derivatives accounted for 10% (*n* = 6) and 7% (*n* = 4) of these inversely associated metabolites, respectively. Out of 42 identified individual metabolites positively associated with CRC, 28 (67%) were lipids and lipid-like molecules. The rest included organic acids and derivatives, organoheterocyclic compounds (each accounting for 14 and 12%, respectively). The remaining categories included nucleosides, nucleotides and their analogs, organic oxygen compounds, and benzenoids (each accounting for 2%, *n* = 1). While the lipids and lipid-like molecules with the positive association were mainly bile acids and fatty acylcarnitines, inverse associations were seen with alkylacyl-lysophosphatidylcholines, phosphatidylcholines, and sphingomyelins. Among the wide range of metabolites identified, only a select few appeared in more than one study. Specific plasma bile acids, including glycocholic acid, taurocholic acid, glycochenodeoxycholic acid, taurochenodeoxycholic acid, glycodeoxycholic acid, and taurodeoxycholic acid, were reported in two large cohort studies to be positively associated with CRC. These bile acids were noted by Kühn et al.^26^ in the EPIC cohort focusing on colon cancer, and by Loftfield et al. ^16^ in the PLCO cohort, with Loftfield et al.^16^ reporting these findings specifically in women. Similarly, amino acids such as valine and tryptophan were identified in multiple studies, though the direction of their associations with CRC varied. Tryptophan was positively associated with CRC in findings by Vidman et al.^24^, while two cohorts studied by Papadimitriou et al.^27^ showed a reverse trend. For the CRC precursors, significant inverse and positive associations were reported for four (i.e., C18:2-c linoleic acid, glycine, C36:3 phosphatidylcholine plasmalogen, and phenylacetylglutamine) and five metabolites (i.e., omega-6 polyunsaturated fatty acid, trans-fatty acid, methionine sulfoxide/methionine ratio, C18:1 sphingomyelin, and C54:8 triglyceride), respectively, of which two and four metabolites belonged to the group of lipids and lipid-like molecules. The three other metabolites belonged to the group of organic acids and derivatives.

**Table 2:** Individual metabolites associated with the presence or occurrence of CRC in blood, urine, and stool samples

First author Year	Platform	Biospecimen	Number of metabolites identified/ Metabolite identification approach^c^	Outcome	Associated metabolites with outcome ^a^	
					Inverse association	Positive association
**Screening**						
Pickens (2017) ^35^	GC	Plasma	24 fatty acids	A ♂	-	ω−6 polyunsaturated fatty acid Trans-fatty acid
				HPP ♂	C18:2-c linoleic acid	-
Tevini (2022) ^37^	FIA and LC-MS/MS	Serum	188 AbsoluteIDQ® p180 kit	AA	Glycine	methionine sulfoxide/methionine ratio SM C18:1
				CRC	*Glycerophospholipids* (LysoPC a C17:0, LysoPC a C18:0, LysoPC a C18:2, LysoPC a C26:0, LysoPC a C28:0, LysoPC a C28:1, PC aa C28:1, PC aa C30:0, PC aa C32:2, PC aa C32:3, PC aa C34:3, PC aa C34:4, PC aa C36:2, PC aa C36:6, PC aa C38:0, PC aa C38:1, PC aa C42:6, PC ae C30:0, PC ae C34:0, PC ae C34:2, PC ae C34:3, PC ae C36:1, PC ae C36:2, PC ae C36:3, PC ae C38:0, PC ae C38:3, PC ae C40:1, PC ae C40:6) *Sphingomyelins* (SM (OH) C22:1,SM (OH) C22:2, SM (OH) C24:1, SM C16:1) Histidine Total AC-DC/Total AC Total PC ae Total SM (OH) Total SM (OH)/ total SM (non-OH)	*Acylcarnitines* (C7-DC, C12, C12:1, C14:1, C16:2, C18:1)
**Cohorts**						
Hang (2022) ^34^	LC–MS	Plasma	207 Untargeted	A ♀	C36:3 phosphatidylcholine plasmalogen	
				SP ♀	Phenylacetylglutamine	C54:8 triglyceride
Kühn (2020) ^26^	LC–MS	Plasma	17 Bile acids	CC		Glycocholic acid Taurocholic acid Glycochenodeoxycholic acid Taurochenodeoxycholic acid Glycohyocholic acid Glycodeoxycholic acid Taurodeoxycholic acid
Papadimitriou (2021) ^27^	LC–MS	Plasma	3 Tryptophan metabolites	CC	Tryptophan Kynurenine	Kynurenine Serotinin Kynurenine−to−tryptophan ratio
Cai (2006) ^12^	LC–MS	Urine	1 Prostaglandin E2 Metabolite (PGE-M)	CRC, CC, RC ♀	PGE-M	
Cross (2014) ^13^	LC–MS and GC-MS	Serum	278 Untargeted	CRC	^- b^	
Kühn (2016) ^15^	LC-MS/MS and FIA-MS/MS	Plasma	120 MetaDisIDQTM Kit	CRC	^- b^	
Myte (2017) ^18^	LC-MS/MS and GC-MS, *Lactobacillus casei* and *Lactobacillus leichmannii*	Plasma	14 One-carbon metabolites	CRC	Riboflavin Ppyridoxal 5-phosphate	
Shu (2018) ^21^	UPLC-QTOFMS and GC-TOFMS	Plasma	167 Untargeted	CRC	2-methyl-4-phenyl-2-butyl 2-methylpropanoate PE(20:0/18:2) PC(22:6/18:0) Ethyl 4-(methylthio)butyrate PE(p-16:0/20:4) 5,6–8,9-diepoxyergost-22-ene-3,7beta-diol	Picolinic acid Selenocystine 2,3-epoxymenaquinone
Geijsen (2019) ^14^	UHPLC-QTOF-MS	Plasma	28 Untargeted	CRC	LysoPC(16:1) LysoPC(P-16:0) LysoPC(15:0) LysoPC(16:0) LysoPC(16:0) isomer LysoPC(17:0) LysoPC(18:0) Leucine Valine Bilirubin 1-Methylnicotinamide	LysoPE(20:4) LysoPE(22:6) Taurine Hypoxanthine
McCullough (2021) ^17^	LC-MS/MS	Plasma	886 Untargeted	CRC	3-methylxanthine	Guanidinoacetate Vanillylmandelate 2’-*O*-methylcytidine Bilirubin (E-E) *N*-palmitoylglycine
Loftfield (2022) ^16^	LC-MS/MS	Serum	21 Bile acids and short-chain fatty acids	CRC ♀	-	Glycochenodeoxycholic acid Taurochenodeoxycholic acid Glycocholic acid Taurocholic acid Deoxycholic acid Glycodeoxycholic acid Taurodeoxycholic acid Glycholithocholic acid Taurolithocholic acid
				CRC ♂	Cholic acid	-
Pham (2022) ^19^	ELISA assays	Serum	1 Resistin	CRC	^- b^	
Vidman (2023) ^24^	LC–MS	Plasma	5015 Untargeted	CRC	Sebacic acid Pyroglutamic acid Hydroxytigecycline	9,12,13-TriHOME 13-OxoODE Valine 3-hydroxybutyric acid l-tryptophan

*GC* gas chromatography, *LC-MS/MS* liquid chromatography–mass spectrometry/liquid chromatography/ tandem mass spectrometry, *FIA* flow injection analysis, *GC-MS* gas chromatography–mass spectrometry, *FIA-MS/MS* flow injection analysis–tandem mass spectrometry, *UPLC-QTOFMS* ultra-performance liquid chromatography quadrupole-time-of-flight mass spectrometry, *GC-TOFMS* gas chromatography time-of-flight mass spectrometry, *UHPLC-QTOF-MS* ultra-high chromatography- quadrupole-time-of-flight mass spectrometry, *AC* acylcarnitine, *LysoPC* monoacyl-glycerophosphocholine, *PC* *aa* diacyl-glycerophosphocholine, *PC ae* alky-acyl-glycerophosphocholine, *SM* sphingomyelin, *AC* acylcarnitine, *(A)A* (advanced) adenoma, *AP* clonic adenomatous polyps, *Cn* controls, *CC*, colon cancer, *RC* rectal cancer, *CRC* colorectal cancer, *SP* serrated polyps, *HPP* hyperplastic polyps, ♀ female, ♂ male.

^a^Includes only named metabolites.

^*b*^No significant associations with metabolites identified (after correction for multiple testing).

^c^Describes the metabolite identification method used: targeted groups, untargeted approaches, or specific commercial panels.

Out of 15 studies that examined metabolites individually, only three conducted internal validation^18,24,37^ and two performed external validation^14,27^. Papadimitriou et al. ^27^ examined three metabolites of tryptophan in three different samples. However, they found inconsistent directions of association for two of the metabolites, tryptophan and kynurenine, and their ratio, between the three studies. Geijsen et al. ^14^ applied an untargeted approach and identified 15 metabolites that differed significantly between cases and controls of CRC in both their discovery and replication sets. However, whether these metabolites were of predictive or prognostic value was not identified. Except for the studies by Tevini et al. ^37^ and Cai et al. ^12^, all the other studies that investigated metabolites individually adjusted for several covariates in their analyses, such as age, smoking status, or BMI (see Supplementary Table 5).

Tables 3 and 4 present the metabolite biomarker panels developed for detection CRC and its precursors. Among the 13 studies that reported on these panels, one conducted an external validation^31^, while eight performed internal validations^22,23,28–30,32,33,36^. The remaining four studies did not conduct any form of validation^16,20,21,25^.

**Table 3:** Performance characteristics of metabolite panels to predict the presence or occurrence of CRC in blood biospecimen

First author Year	Biospecimen	Platform	Outcome	Metabolite panel	Performance			
					OR (95% CI)	AUC	Sensitivity (%)	Specificity (%)
**Screening**								
Liu (2023) ^29^	Serum	IL-MS	A	Glutamine, Threonine		0.83^d^		
				Asparagine, Glutamine, Threonine		0.85^d^		
				Arginine, Asparagine, Glutamine, Threonine		0.87^d^		
Troisi (2022) ^23^	Serum	GC-MS	CRC	Acetic, Androstenedione, Aspartic, Estradiol, Fructose, Glucose, Glutamine, Guanine, Hydroxylamine, Isoleucine, Lactose, Myristic, Nicotinic, Norepinephrine, Oxalic, Oxoglutaric, Oxoproline, Propionic, Pyrocatechol, Pyruvic, Quinolinic, Tartaric, Tetra, Threonine, Urea, Valine		1.0^d^	100^d^	100^d^
**Cohorts**								
Farshidfar (2016) ^28^	Serum	FIA-MS/MS	A	Decenoylcarnitine, Dodecenoylcarnitine, Hexadecadienylcarnitine, Hydroxytetradecenoylcarnitine, lysoPhosphatidylcholine acyl C17:0, Phosphatidylcholine acly-alkyl C40:2, Proline, Tetradecadienylcarnitine, Tryptophan		0.82^d^		
Shu (2018) ^21^	Plasma	UPLC-QTOFMS and GC-TOFMS	CRC	2,3-epoxymenaquinone, 2-methyl-4-phenyl-2-butyl 2-methylpropanoate, 5,6:8,9-diepoxyergost-22-ene-3,7beta-diol, Ethyl 4-(methylthio)butyrate, PC(22:6/18:0), PE(20:0/18:2), PE(p-16:0/20:4), Picolinic acid, Selenocystine		0.76		
Loftfield (2022) ^16^	Serum	LC-MS/MS	CRC ♀	Chenodeoxycholic acid, Cholic acid, Deoxycholic acid, Glycochenodeoxycholic acid, Glycocholic acid, Glycodeoxycholic acid, Glycolithocholic acid, Glycoursodeoxycholic acid, Lithocholic acid, Taurochenodeoxycholic acid, Taurocholic acid, Taurodeoxycholic acid, Taurolithocholic acid, Ursodeoxycholic acid	1.95 (1.04, 3.66)^a^			
				Acetic acid, Butyric acid, Hexanoic acid, Isobutyric acid, Isovaleric acid, Propionic acid	0.55 (0.31, 0.98)^a^			
Rothwell (2022) ^20^	Serum and plasma	GC and LC-MS/MS	CRC	2:1n-9, 15:0, 15:01, 16:00, 16:1n-7/n-9, 17:0, 18:1n-9c, 20:3n-9, 22:5n-6	0.51 (0.29, 0.90)^b^			
			CC		0.53 (0.29, 0.97)^b^			
			CRC	Glycine, Glutamate, lysoPC a C17:0, lysoPC a C18:2, PC aa C32:1, PC aa C34:4, PC aa C36:4, PC aa C38:4, PC aa C40:4, PC ae C36:2, PC ae C38:2, PC ae C38:3, PC ae C40:6, Serine	0.62 (0.50, 0.78)^c^			
			CC		0.65 (0.50, 0.84)^c^			
			RC		0.44 (0.25, 0.79)^c^			

*LC-MS/MS* liquid chromatography–mass spectrometry, *GC-MS* gas chromatography–mass spectrometry, *FIA-MS/MS* flow injection analysis–tandem mass spectrometry, *IL-MS* isobaric labeling mass spectrometry, *GC-TOFMS* gas chromatography time-of-flight mass spectrometry, *UPLC-QTOFMS* ultra-performance liquid chromatography quadrupole-time-of-flight mass spectrometry, *OR* odds ratio, *CI* confidence interval, *AUC* area under the curve, *A* adenoma, *Cn* Controls, CC colon cancer, *CRC* colorectal cancer, ♀ female, ♂ male.

^a^OR comparing highest versus lowest quartile;

^b^OR per unit increase;

^c^OR per unit change;

^d^Internally validated results.

**Table 4:** Performance characteristics of metabolite panels to predict the presence or occurrence of CRC in stool and urine biospecimen

First author Year	Biospecimen	Platform	Outcome	Metabolite panel	Performance		
					AUC	Sensitivity (%)	Specificity (%)
**Screening**							
Wang (2014) ^33^	Urine	NMR	AP	2-Oxoglutarate, 3-Hydroxybutyrate, 3-Hydroxyphenylacetate, 3-Hydroxymandelate, Acetone, Adipate, Asparagine, b-Alanine, Benzoate, Butyrate, Ethanol, Histidine, Methanol, p-Methylhistidine, Serine, Trigonelline, Tyrosin	-	82.7^b^	51.2^b^
Deng (2017a) ^30^	Urine	LC-MS/MS	AP	Ascorbic acid, Carnitine, Succinic Acid	0.69^b^	82.4^b,c^	36.0^b,c^
Deng (2017b) ^31^	Urine	NMR	AP	Ascorbic acid, Carnitine, Succinic Acid	0.72^b^	82.6^b^	42.4^b^
Eisner (2013) ^32^	Urine	^1^H-NMR	P	Acetone, Methanol, Trigonelline, Tyrosine	0.72^a^	64.0^a^	65.0^a^
Telleria (2022) ^22^	Stool	UPLC-MS/MS	AA	Bilirubin E,E, Glycocholenate sulfate, Lactosyl-N-palmitoyl-sphingosine, STLVT	0.95^b^	70.0^b^	100^b^
Amiot (2015) ^36^	Stool	^1^H-NMR	AA/ CRC	Valerate, Butyrate, Propionate, Acetate, Glutamate, Glutamine, ß-Glucose	0.94^b^		
Xie (2023) ^25^	Stool	UPLC‑MS/MS	CRC	9,10‑dihydroxy‑12‑octadecenoic acid, cholesterol ester (18:2), lipoxinA4	0.97		

*NMR* nuclear magnetic resonance spectrometry, *LC-MS/MS* liquid chromatography–mass spectrometry, ^1^*H-NMR* proton nuclear magnetic resonance, *UPLC-MS/MS* ultra-high-performance liquid chromatography–tandem mass spectroscopy, *AUC* area under the curve, *AA* advanced adenoma, *AP* colonic adenomatous polyps, *Cn* controls, *CRC* colorectal cancer, *P* polyps.

^a^Externally validated results,

^b^Internally validated results,

^c^Different sensitivities and specificities available according to threshold criteria in the publication.

Table 3 displays the efficacy of blood-based biomarker panels, with the most effective panel achieving an AUC of 1.0, and 100% sensitivity and specificity^23^. This panel consisted of 26 metabolites and used a machine-learning approach.

Table 4 shows the metabolite biomarkers from stool and urine samples. It includes three studies that analyzed stool sample panels, reporting AUCs of 0.95^22^, 0.94^36^, and 0.97^25^, with the latter not performing any kind of validation. The panel by Telleria et al. further reported a sensitivity of 70% and specificity of 100%, using four metabolites and levels of hemoglobin to discriminate between cases and non-cases of advanced adenoma^22^. For the panels of metabolites based on urine, all studies performed either internal or external validation. The study by Wang et al. ^33^ showed the highest AUC of 0.752, along with sensitivity of 88.9% and specificity of 50.2% for a panel composed of 18 metabolites to distinguish between polyp cases and non-cases. The internal validation confirmed these results, with a sensitivity of 82.7% and a specificity of 51.2%. Deng et al. ^31^ conducted an external validation of a urine-based diagnostic panel for the detection of adenomatous polyps, that was originally developed and validated using *n* = 1000 samples from a Canadian cohort^30^. The external validation in the Chinese cohort yielded an AUC of 0.72, a sensitivity of 82.6%, and a specificity of 42.4%. The panel consisted of four metabolites in combination with information on the age, sex, and smoking status of the participants.

### Quality assessment of diagnostic accuracy studies

In this study, we utilized the QUADAS-2 tool to evaluate the risk of bias and applicability concerns. Detailed results are provided in Supplementary Table 4. For the “patient selection” domain, two studies were identified with a high risk of bias due to small case numbers and large significant differences between cases and controls, while 16 indicated a low risk, and eight were unclear. In the ‘index test’ domain, the risk of bias was low in ten studies, unclear in 15, and high in one. Similarly, for the ‘reference standard’ domain, the risk assessment showed 16 studies with low risk, ten with unclear risk, and none with high risk. The unclear risk assessments in the “index test” and “reference standard” domains were partly due to the absence of information in some studies about the independent execution of metabolite tests and their comparison counterparts, such as colonoscopies. In the “flow and timing” domain, eight studies were assessed as low risk, eight as high risk, and ten as unclear. Predominantly, the studies were highly applicable, a result of our focused method in selecting articles pertinent to CRC or its early predictors. Nonetheless, we observed significant applicability issues in “patient selection” for ten studies, mainly because of missing internal or external validation and a narrow demographic focus. There were no applicability concerns for the “index test” and predominantly no in “reference standard” domains, as these tests align with our review question.

## Discussion

In the present systematic review, we identified 26 studies focusing on metabolite biomarkers for the prediction of the occurrence or presence of CRC or its precursors. These studies contributed valuable insights into metabolomics within the context of CRC screening trials and prospective cohort studies. Lipids and lipid-like molecules emerged as the most frequently investigated metabolites across various biospecimens, offering the potential for CRC and its precursors prediction in the context of CRC screening or risk assessment. However, the heterogeneity in data analysis methodologies and result reporting hindered a unified interpretation and precluded a meta-analytic approach. Specifically, this variability in the use of different metabolite panels, statistical models, and validation techniques limits comparability and introduces challenges in synthesizing data across studies. Therefore, a descriptive presentation of findings was conducted. Additionally, most studies showed a lack of robust validation for their biomarker panels, often only performing internal validation, which questions the generalizability of the findings. The small sample sizes in several studies further constrained the statistical power, increasing the risk of erroneous results. A notable geographical bias toward white and Asian populations was also observed, affecting the applicability of findings to other ethnic groups. While individual studies displayed advancements in metabolomics profiling, the absence of consistent validation across studies underscores the need for standardized methodological frameworks in future research.

The comparison between individual metabolites and metabolite panels reveals a notable pattern, suggesting that the latter holds superior potential as a screening tool or risk assessment tool for CRC screening. Three out of 15 studies^13,15,19^ based on individual metabolites did not find any significant associations after correcting for multiple testing. In contrast, studies examining metabolite panels consistently demonstrated good to very good predictive or diagnostic abilities. This observation, supported by a systematic review incorporating also post-diagnostic metabolite samples^11^, suggests that metabolite panels may possess the capacity to better reflect the complexity of biological systems, address disease heterogeneity, and offer synergistic insights into collective metabolic alterations associated with CRC development, unlike individual metabolites.

Notably, a range of panels have yielded exceptionally high AUC values between 0.76 and an optimal 1.0 for CRC detection or prediction, with AUCs exceeding 0.83 for early indicators of CRC, with some consisting of merely two metabolites^29^, while others included up to 26 metabolites^23^. However, these high-performance panels, in some instances, were evaluated in studies utilizing relatively small sample sizes of fewer than 50 cases^25,28,29,36,37^ and were only examined in a single population. While more than half of the studies implemented internal validation, predominantly using split-sampling methods for model testing, only three studies undertook external validation^14,27,31^. These studies revealed varied outcomes: certain metabolites displayed unreliable or minimal correlations with CRC in diverse populations, whereas others achieved results on par with current stool tests. While Gejisen et al. ^14^ replicated their untargeted approach findings, revealing 15 metabolites significantly associated with CRC in two European cohorts, Papadimitriou et al. ^27^ reported inconsistent associations between tryptophan metabolism-linked metabolites and colon cancer across cohorts. Deng et al. achieved comparable metabolite test performance in the studied Chinese cohort to the original Canadian cohort in which the metabolite panel was developed^30,31^. While this panel exhibited increased sensitivity, its specificity was somewhat lower compared to well-established fecal blood tests that have specificities for advanced adenomas ranging from 0.90 to 0.95^38^. These varied outcomes point to a significant challenge in the field of metabolite biomarker research, emphasizing the critical need for thorough independent validation^39^. The importance of such validation is heightened by the fact that metabolite stability can differ over time and with various sample collection methods^40^. Thorough independent validation is essential to mitigate the risk of overestimating predictive capabilities, often referred to as the “winner’s curse”, where models may appear highly predictive in initial derivation but fail to perform as well in subsequent applications. Internal validation helps address this by proper evaluation of the model within the same dataset (e.g., by a split sample or cross-validation approaches), reducing the likelihood of overfitting. External validation not only confirms the robustness of these findings but also identifies potential limitations in different demographic or clinical settings, ensuring that the predictive models can be reliably applied in various real-world scenarios.

Several metabolic pathways, including glycolysis, glutaminolysis, oxidative phosphorylation, and lipid metabolism^41^, appear to be altered during the cancer state. Notably, lipid metabolism stands out, as lipids and lipid-like molecules frequently emerge as the most altered metabolites in CRC risk prediction. Among these, two studies identified elevated levels of plasma bile acids, including glycocholic acid, taurocholic acid, glycochenodeoxycholic acid, taurochenodeoxycholic acid, glycodeoxycholic acid, and taurodeoxycholic acid, to be positively associated with CRC^16,26^. These bile acids may contribute to carcinogenesis through their roles in disrupting cell signaling pathways, promoting inflammation, and inducing DNA damage in colorectal epithelial cells^42,43^. Additionally, bile acids can activate nuclear receptors, which are involved in lipid metabolism, cellular proliferation, and apoptosis regulation^42^. This may reflect their vital roles in cellular functions essential for cancer development, such as cell membrane integrity, energy storage, and signaling^44,45^. Additionally, the prevalence of lipids in these findings could also be influenced by their prominence in commercially available metabolomics kits and the specific research focus on these molecules, which may skew the observed metabolic alterations toward lipid-related pathways. Further, the precise timing of these metabolic changes remains unclear, underscoring a significant area for future research to explore the temporality of metabolite biomarker alterations in the context of cancer progression. Research from screening trials and nested case-control studies within prospective cohorts provides a unique opportunity to investigate the temporality of metabolite biomarker performance. In nested case-control studies and prospective cohort studies, where samples are collected on average several years before diagnosis, risk-predictive biomarkers gain importance. For example, these biomarkers hold the potential for application in individuals before the starting age for screening, facilitating risk assessment, and the development of more refined risk prediction algorithms. Current risk-prediction algorithms, incorporating factors such as age, family history, genetic risk factors, and lifestyle factors, show promise but require further improvement^46^. Conversely, metabolite biomarkers identified in screening trials, shortly before the diagnosis of CRC or its precursors, may provide valuable insights for refining and optimizing diagnostic strategies, leveraging the screening trials’ capability to capture biomarkers indicative of the imminent occurrence of CRC.

Consideration should also be given to the temporal aspect related to the stage of colorectal carcinogenesis examined in the selected studies. Metabolite profiles may exhibit distinct patterns at various stages of CRC progression, with specific metabolites associated with aggressive tumor characteristics being more pronounced in CRC compared to adenomas or polyps^47^. Recognizing and leveraging these nuanced metabolic panels could enhance the accuracy of metabolite-based diagnostics, enabling more precise differentiation between CRC, adenomas, and polyps.

Metabolites, integral to the phenotype, are extractable from diverse biospecimens, including blood, urine, and stool, with blood and urine being the most common choices in the examined studies. The results based on different biospecimens are only partly comparable. Notably, negative correlations have been observed between metabolite concentrations in stool and urine samples, whereas positive correlations exist between blood and urine, as well as blood and stool metabolite concentrations^48^. Tumor-related detection of metabolites in blood samples, which are routinely collected in medical practice, exhibits challenges with indirect tumor analysis and potential analyte dilution from leaked cells^49^. Conversely, metabolites derived from urine and stool samples show promise in capturing CRC-related metabolic perturbations, potentially reflecting the tumor microenvironment^50^. In contrast to the complexity of blood analysis, the simplicity of urine and stool provides unique advantages. However, variations in metabolite concentrations due to circadian rhythm and diet necessitate standardizing collection time and controlling for nutrient consumption patterns^51^. Especially concentrations of fatty acids, lipids, and amino acids are known to show circadian variation^52^. Additionally, metabolite concentrations depend on whether a person is fasting or has recently eaten, with decreases in acylcarnitine and triglycerides and increases in amino acids and glucose-related metabolites after a meal^52^.

The inclusion of various sets of covariates adds to the complexity of comparing the performance of different individual metabolites and metabolite panels across the studies. Age, sex, and various clinical variables were included as covariates in the models, with age and sex being the most frequently integrated factors. However, many metabolites are affected by lifestyle and nutritional factors and are subject to temporal variation caused by such factors^53,54^. Standardized conditions of sample collection, along with careful ascertainment of potential non-tumor related determinants is crucial for establishing potential use of metabolomics in CRC risk assessment or early detection^55^.

Metabolite identification is subject to significant variation due to the varied use of analytical techniques, technical implementation, and the use of various techniques of data analyses across the included studies. The choice of analytical techniques, such as NMR, GC-MS, and LC–MS, introduces distinctive approaches to metabolite identification. NMR, as the most popular option, offers the possibility to detect a wide range of metabolites, while alternative methods like ELISA assays offer enhanced flexibility, demonstrating the diverse spectrum of tools available. Technical factors also play a crucial role in the variation of the metabolite identification. The time and temperature of sample collection and freezing can significantly influence outcomes. Standardizing protocols for sample collection, pre-analytical handling, and storage conditions is essential to minimize variations, ensuring reproducibility in metabolomics research^55^. Likewise, initiatives to standardize metabolomics analyses are crucial in this regard, as they aim to establish consistent protocols across studies^55,56^. These include guidelines for study design, sample processing, and data reporting, which are necessary to reduce inconsistencies and improve the comparability of results across different laboratories and studies^55^.

In parallel, the integration of various techniques of statistical analysis, exemplified by the LASSO algorithm and Bayesian network in the included studies^18,23,30,32^, introduces another layer of complexity. These techniques prove valuable in identifying metabolites that differentiate between CRC or precursor cases and controls. The combination of metabolomics and machine learning offers an alternative to traditional statistical methods, particularly for addressing the challenges presented by non-linear biological data^57^.

The direct comparison of the results obtained for the identified metabolite panels and for the individual metabolites is complicated by a variety of factors, such as differing analytical methods and technical considerations. The potential introduction of metabolomics testing in clinical practice should be accompanied by careful evaluation of cost-effectiveness studies. So far, cost-effectiveness studies have been very limited. One such study concluded that implementing urine-based metabolomics tests, such as those from Deng et al. ^30,31^, might be a cost-effective strategy in programmatic CRC screening programs^58^. Therefore, the translation of these findings into clinical practice is not imminent, highlighting the need for careful consideration of the complex intricacies involved.

A strength of our review is its sole focus on studies where biospecimens were collected before diagnosis of CRC or CRC precursors, differentiating it from most metabolomics research based on samples collected after diagnosis, whose relevance for early detection remains uncertain. Additionally, the review’s comprehensive approach, covering a broad spectrum of metabolite biomarkers in various biospecimens, improves our understanding of CRC metabolomics, potentially unlocking new insights into CRC prediction and risk assessment.

Limitations in the interpretation and implementation of metabolomics studies pose challenges. A major concern is the lack of standardization, with efforts from initiatives aimed at establishing standardized protocols from study design to sample collection and preparation^55^. This lack of standardization may hinder the comparability of studies included in this systematic review. While the review provides a narrative summary, it does not include a meta-analysis due to the heterogeneity of the studies. This decision, while justified in light of the lack of standardization, means that the review does not offer a quantitative synthesis of the data, which could potentially yield more definitive conclusions. Furthermore, this systematic review faces potential challenges beyond those inherent to the included studies, such as publication bias, and the variability and sometimes insufficient detail in the data reported by the individual study publications.

This systematic review emphasizes the significant potential of metabolite panels, particularly those that focus on lipids, in improving CRC prediction and risk assessment, outperforming the accuracy of individual metabolites. These panels, based on metabolites derived from blood, urine, and stool samples, have the potential to enhance CRC screening by enabling accurate risk assessment, thereby optimizing resource allocation, and identifying individuals at high risk. However, the variation in analytical methods and the lack of a standardized validation process underscore the need for methodological harmonization. By standardizing techniques, ensuring thorough validation, and examining metabolic variations at different CRC stages, metabolomics might have the potential to be effectively incorporated into clinical practice, potentially transforming CRC screening strategies to align with the emerging focus on personalized and precision medicine.

## Methods

Our systematic review was conducted following a pre-registered study protocol with PROSPERO (registration number: CRD42023425862). Any modifications made during the review process were documented in PROSPERO to ensure transparency and consistency. Additionally, we adhered to the standardized methodology guidelines outlined in the Preferred Reporting Items for Systematic Reviews and Meta-Analyses (PRISMA) (Supplementary Table 1)^59^.

### Search strategy

Our systematic literature search aimed to identify relevant studies focusing on metabolite biomarkers in noninvasive (urine, stool) or minimally invasive (blood) biospecimens analyzed in pre-diagnostic settings, concentrating on CRC or its precursors. The search was conducted on December 30, 2023, across three electronic databases, including PubMed, Web of Science, and Scopus. The search terms employed consisted of “metabolomics”, “pre-diagnostic biomarker”, and “colorectal cancer” along with associated terms. Details regarding the employed terms for each database are available in Supplementary Table 2.

### Study selection

In our selection process, we considered articles on studies conducted in a screening context that involved the measurement of metabolomics in biospecimens (blood, urine, or stool) taken before a diagnosis of CRC or its precursors. Additionally, we included articles based on prospective cohort studies in which metabolomics measurements were obtained from biospecimens collected at baseline. The primary outcome of interest encompassed CRC, its anatomic subsites (rectal or colon cancer), or precursors such as adenomas or polyps. Letters, editorials, comments, news articles, or articles published in languages other than English were not included. Records unrelated to our review question, such as those focusing on different cancer types or biospecimen collection after diagnosis, were also excluded. We furthermore excluded records that lacked sufficient statistical data or did not report on the diagnostic or predictive performance of metabolite biomarkers.

### Data extraction and evaluation of study quality

Data extraction was performed independently by two authors, TS and CF. To ensure precision and reliability, any initial discrepancies were resolved through consensus after a thorough review and discussion. Information extracted from each study included publication details (e.g., first author, publication year), population characteristics (country, study design, study setting, sample size, mean or median age of participants, and proportion of female participants), sample characteristics (type of biospecimen, technique used for metabolomics analysis, and the specific metabolites evaluated), as well as effect measures, statistical methods, and study results, such as the diagnostic or predictive performance of the studied metabolite biomarkers.

The methodological quality of each record was independently assessed by two investigators, TS and CF, using the Quality Assessment of Diagnostic Accuracy Studies 2 (QUADAS-2) tool^60^. Initial disagreements were resolved through consensus after further review and discussion. The assessment of risk of bias included four domains: “patient selection,” “index test,” “reference standard,” and “flow and timing,” and the evaluation of applicability comprised three domains: “patient selection”, “index test”, and “reference standard”. The risk of bias and applicability assessment for each study was rated as “high risk/concern,” “low risk/concern,” or “unclear risk/concern” based on the QUADAS-2 signaling questions^60^.

## Supplementary information

Supplementary Material

## Data Availability

All data generated and analyzed during this study are included in the article and its supplementary information files.
