# Supplementary material for: Potential of pre-diagnostic metabolomics for colorectal cancer risk assessment or early detection
Source: NPJ Precis Oncol. 2024 Oct 27;8:244. doi: 10.1038/s41698-024-00732-5 (PMC11514036; doi:10.1038/s41698-024-00732-5)
Supplement: Supplementary file 1 — Supplementary Material [file 41698_2024_732_MOESM1_ESM.pdf]

Supplementary Table 1. Preferred Reporting Items for Systematic Reviews and Meta-Analyses (PRISMA) checklist

Section and Topic	Item #	Checklist item	Location
**TITLE**			
Title	1	Identify the report as a systematic review.	Title page
**ABSTRACT**			
Abstract	2	See the PRISMA 2020 for Abstracts checklist.	P1
**INTRODUCTION**			
Rationale	3	Describe the rationale for the review in the context of existing knowledge.	P1
Objectives	4	Provide an explicit statement of the objective(s) or question(s) the review addresses.	P1
**METHODS**			
Eligibility criteria	5	Specify the inclusion and exclusion criteria for the review and how studies were grouped for the syntheses.	P11
Information sources	6	Specify all databases, registers, websites, organisations, reference lists and other sources searched or consulted to identify studies. Specify the date when each source was last searched or consulted.	P11
Search strategy	7	Present the full search strategies for all databases, registers and websites, including any filters and limits used.	S.Table 2
Selection process	8	Specify the methods used to decide whether a study met the inclusion criteria of the review, including how many reviewers screened each record and each report retrieved, whether they worked independently, and if applicable, details of automation tools used in the process.	P11
Data collection process	9	Specify the methods used to collect data from reports, including how many reviewers collected data from each report, whether they worked independently, any processes for obtaining or confirming data from study investigators, and if applicable, details of automation tools used in the process.	P11
Data items	10a	List and define all outcomes for which data were sought. Specify whether all results that were compatible with each outcome domain in each study were sought (e.g. for all measures, time points, analyses), and if not, the methods used to decide which results to collect.	P11
	10b	List and define all other variables for which data were sought (e.g. participant and intervention characteristics, funding sources). Describe any assumptions made about any missing or unclear information.	P11
Study risk of bias assessment	11	Specify the methods used to assess risk of bias in the included studies, including details of the tool(s) used, how many reviewers assessed each study and whether they worked independently, and if applicable, details of automation tools used in the process.	P11
Effect measures	12	Specify for each outcome the effect measure(s) (e.g. risk ratio, mean difference) used in the synthesis or presentation of results.	P5
Synthesis methods	13a	Describe the processes used to decide which studies were eligible for each synthesis (e.g. tabulating the study intervention characteristics and comparing against the planned groups for each synthesis (item #5)).	-

	13b	Describe any methods required to prepare the data for presentation or synthesis, such as handling of missing summary statistics, or data conversions.	-
	13c	Describe any methods used to tabulate or visually display results of individual studies and syntheses.	-
	13d	Describe any methods used to synthesize results and provide a rationale for the choice(s). If meta-analysis was performed, describe the model(s), method(s) to identify the presence and extent of statistical heterogeneity, and software package(s) used.	-
	13e	Describe any methods used to explore possible causes of heterogeneity among study results (e.g. subgroup analysis, meta-regression).	-
	13f	Describe any sensitivity analyses conducted to assess robustness of the synthesized results.	-
Reporting bias assessment	14	Describe any methods used to assess risk of bias due to missing results in a synthesis (arising from reporting biases).	-
Certainty assessment	15	Describe any methods used to assess certainty (or confidence) in the body of evidence for an outcome.	-
**RESULTS**			
Study selection	16a	Describe the results of the search and selection process, from the number of records identified in the search to the number of studies included in the review, ideally using a flow diagram.	Fig 1
	16b	Cite studies that might appear to meet the inclusion criteria, but which were excluded, and explain why they were excluded.	-
Study characteristics	17	Cite each included study and present its characteristics.	Table 1
Risk of bias in studies	18	Present assessments of risk of bias for each included study.	S. Table 4
Results of individual studies	19	For all outcomes, present, for each study: (a) summary statistics for each group (where appropriate) and (b) an effect estimate and its precision (e.g. confidence/credible interval), ideally using structured tables or plots.	Table 2-4
Results of syntheses	20a	For each synthesis, briefly summarise the characteristics and risk of bias among contributing studies.	-
	20b	Present results of all statistical syntheses conducted. If meta-analysis was done, present for each the summary estimate and its precision (e.g. confidence/credible interval) and measures of statistical heterogeneity. If comparing groups, describe the direction of the effect.	-
	20c	Present results of all investigations of possible causes of heterogeneity among study results.	-
	20d	Present results of all sensitivity analyses conducted to assess the robustness of the synthesized results.	-
Reporting biases	21	Present assessments of risk of bias due to missing results (arising from reporting biases) for each synthesis assessed.	-
Certainty of evidence	22	Present assessments of certainty (or confidence) in the body of evidence for each outcome assessed.	-
**DISCUSSION**			
Discussion	23a	Provide a general interpretation of the results in the context of other evidence.	P7
	23b	Discuss any limitations of the evidence included in the review.	P9/10

	23c	Discuss any limitations of the review processes used.	P9/10
	23d	Discuss implications of the results for practice, policy, and future research.	P9/10
**OTHER INFORMATION**			
Registration and protocol	24a	Provide registration information for the review, including register name and registration number, or state that the review was not registered.	P10
	24b	Indicate where the review protocol can be accessed, or state that a protocol was not prepared.	P10
	24c	Describe and explain any amendments to information provided at registration or in the protocol.	Available at PROSPERO
Support	25	Describe sources of financial or non-financial support for the review, and the role of the funders or sponsors in the review.	P12
Competing interests	26	Declare any competing interests of review authors.	P12
Availability of data, code and other materials	27	Report which of the following are publicly available and where they can be found: template data collection forms; data extracted from included studies; data used for all analyses; analytic code; any other materials used in the review.	P12

Supplementary Table 2. Search strategy and results

**PubMed**	#1<br>(colorect*[Title/Abstract] OR colon[Title/Abstract] OR colonic[Title/Abstract] OR rectal*[Title/Abstract] OR rectum*[Title/Abstract] OR "large bowel"[Title/Abstract]) AND (cancer*[Title/Abstract] OR malignan*[Title/Abstract] OR carcinom*[Title/Abstract] OR tumo*[Title/Abstract] OR CRC[Title/Abstract] OR adenocarcinoma*[Title/Abstract] OR adenom*[Title/Abstract] OR lesion*[Title/Abstract] OR neoplas*[Title/Abstract])<br>*343,477 results*
	#2<br>(metabolomics[MeSH Terms] OR metabolome[MeSH Terms] OR metabolite*[Title/abstract] OR metabolo*[Title/abstract] OR metabonom*[Title/abstract] OR metabolite network*[Title/abstract] OR metabolite profile*[Title/abstract] OR lipidom*[Title/abstract])<br>*395,855 results*
	#3<br>(screen*[Title/Abstract] OR cohort[Title/Abstract] OR prospective[Title/Abstract] OR prediagnostic[Title/Abstract] OR pre-diagnostic[Title/Abstract] OR asymptomatic[Title/Abstract] OR detection[Title/Abstract] OR diagnosis[Title/Abstract] OR diagnostic[Title/Abstract])<br>*5,442,088 results*
	#4<br>editorial[Publication Type] OR letter[Publication Type] OR Comment*[Publication Type] OR news[Publication Type]<br>*2,440,529 results*
	#5<br>(animal*[Title] OR "animal experiment"[Title] OR "animal model"[Title] OR "animal tissue"[Title] OR "non human"[Title] OR nonhuman[Title] OR rat[Title] OR rats[Title] OR mice[Title] OR mouse[Title] OR swine[Title] OR porcine[Title] OR murine[Title] OR sheep[Title] OR lambs[Title] OR pig[Title] OR pigs[Title] OR piglet*[Title] OR rabbit*[Title] OR monkey[Title] OR bovine[Title])<br>*2,140,655 results*
	**#1 AND #2 AND #3 NOT #4 NOT #5**<br>***976 results***
**Web of Science**	#1<br>((TS=(((colorect* OR colon OR colonic OR rectal* OR rectum* OR "large bowel") AND (cancer* OR malignan* OR carcinom* OR tumo* OR CRC OR adenocarcinoma* OR adenom* OR lesion* OR neoplas*)))) AND LA=(English)) AND DT=(Article OR Abstract of Published Item OR Early Access OR Reprint)<br>*289,459 results*
	#2<br>((TS=((metabolomics OR metabolome OR metabolite* OR metabolo* OR metabonom* OR metabolite network* OR metabolite profile* OR lipidom*))) AND LA=(English)) AND DT=(Article OR Abstract of Published Item OR Early Access OR Reprint)<br>*391,826 results*
	#3<br>((TS=((screen* OR cohort OR prospective OR prediagnostic OR pre-diagnostic OR asymptomatic OR detection OR diagnosis OR diagnostic))) AND LA=(English)) AND DT=(Article OR Abstract of Published Item OR Early Access OR Reprint)<br>*4,804,881 results*
	#4<br>TI=(animal* OR "animal experiment" OR "animal model" OR "animal tissue" OR "non human" OR nonhuman OR rat OR rats OR mice OR mouse OR swine OR porcine OR murine OR sheep OR lambs OR pig OR pigs OR piglet* OR rabbit* OR monkey OR bovine)<br>*2,786,310 results*
	**#1 AND #2 AND #3 NOT #4**<br>***1,224 results***

**SCOPUS**	#1<br>TITLE-ABS-KEY ((colorect* OR colon OR colonic OR rectal* OR rectum* OR "large bowel") AND (cancer* OR malignan* OR carcinom* OR tumo* OR crc OR adenocarcinoma* OR adenom* OR lesion* OR neoplas*)) AND DOCTYPE (ar)<br>*407,351 results*
	#2<br>TITLE-ABS-KEY ((metabolomics OR metabolome OR metabolite* OR metabolo* OR metabonom* OR metabolite AND network* OR metabolite AND profile* OR lipidom*)) AND DOCTYPE (ar)<br>*48,332 results*
	#3<br>TITLE-ABS-KEY ((screen* OR cohort OR prospective OR prediagnostic OR pre-diagnostic OR asymptomatic OR detection OR diagnosis OR diagnostic)) AND DOCTYPE (ar)<br>*8,773,644 results*
	#4<br>TITLE ((animal* OR "animal experiment" OR "animal model" OR "animal tissue" OR "non human" OR nonhuman OR rat OR rats OR mice OR mouse OR swin OR porcine OR murine OR sheep OR lambs OR pig OR pigs OR piglet* OR rabbit* OR monkey OR bovine))<br>*2,588,753 results*
	**#1 AND #2 AND #3 AND NOT #4 AND (LIMIT-TO (LANGUAGE , "English"))**<br>***284 results***

Supplementary Table 3. Articles excluded from full-text screening

**(A) No longitudinal cohort study or screening trial**
1. Altobelli E, Angeletti PM, Latella G. Role of Urinary Biomarkers in the Diagnosis of Adenoma and Colorectal Cancer: A Systematic Review and Meta-Analysis. J Cancer. 2016;7(14):1984-2004. doi: 10.7150/jca.16244.
2. Bezabeh T, Ijare OB, Nikulin AE, Somorjai RL, Smith IC. MRS-based Metabolomics in Cancer Research. Magn Reson Insights. 2014;7:1-14. doi: 10.4137/MRI.S13755.
3. Bosch S, Berkhout DJ, Ben Larbi I, de Meij TG, de Boer NK. Fecal volatile organic compounds for early detection of colorectal cancer: where are we now? J Cancer Res Clin Oncol. 2019;145(1):223-34. doi: 10.1007/s00432-018-2821-3.
4. Brezmes J, Llambrich M, Cumeras R, Guma J. Urine NMR Metabolomics for Precision Oncology in Colorectal Cancer. Int J Mol Sci. 2022;23(19). doi: 10.3390/ijms231911171.
5. Di Lena M, Porcelli F, Altomare DF. Volatile organic compounds as new biomarkers for colorectal cancer: a review. Colorectal Dis. 2016;18(7):654-63. doi: 10.1111/codi.13271.
6. Erben V, Bhardwaj M, Schrotz-King P, Brenner H. Metabolomics Biomarkers for Detection of Colorectal Neoplasms: A Systematic Review. Cancers (Basel). 2018;10(8). doi: 10.3390/cancers10080246.
7. Gallardo-Gomez M, De Chiara L, Alvarez-Chaver P, Cubiella J. Colorectal cancer screening and diagnosis: omics-based technologies for development of a non-invasive blood-based method. Expert Rev Anticancer Ther. 2021;21(7):723-38. doi: 10.1080/14737140.2021.1882858.
8. Gan X, Wang T, Chen ZY, Zhang KH. Blood-derived molecular signatures as biomarker panels for the early detection of colorectal cancer. Mol Biol Rep. 2020;47(10):8159-68. doi: 10.1007/s11033-020-05838-0.
9. Gold A, Choueiry F, Jin N, Mo X, Zhu J. The Application of Metabolomics in Recent Colorectal Cancer Studies: A State-of-the-Art Review. Cancers (Basel). 2022;14(3). doi: 10.3390/cancers14030725.
10. Harlid S, Gunter MJ, Van Guelpen B. Risk-Predictive and Diagnostic Biomarkers for Colorectal Cancer; a Systematic Review of Studies Using Pre-Diagnostic Blood Samples Collected in Prospective Cohorts and Screening Settings. Cancers (Basel). 2021;13(17). doi: 10.3390/cancers13174406.
11. Hashim NAA, Ab-Rahim S, Suddin LS, Saman MSA, Mazlan M. Global serum metabolomics profiling of colorectal cancer. Mol Clin Oncol. 2019;11(1):3-14. doi: 10.3892/mco.2019.1853.
12. Mallafre-Muro C, Llambrich M, Cumeras R, Pardo A, Brezmes J, Marco S, et al. Comprehensive Volatilome and Metabolome Signatures of Colorectal Cancer in Urine: A Systematic Review and Meta-Analysis. Cancers (Basel). 2021;13(11). doi: 10.3390/cancers13112534.
13. Nannini G, Meoni G, Amedei A, Tenori L. Metabolomics profile in gastrointestinal cancers: Update and future perspectives. World J Gastroenterol. 2020;26(20):2514-32. doi: 10.3748/wjg.v26.i20.2514.
14. Ni Y, Xie G, Jia W. Metabonomics of human colorectal cancer: new approaches for early diagnosis and biomarker discovery. J Proteome Res. 2014;13(9):3857-70. doi: 10.1021/pr500443c.
15. Piras C, Pibiri M, Leoni VP, Cabras F, Restivo A, Griffin JL, et al. Urinary(1)H-NMR Metabolic Signature in Subjects Undergoing Colonoscopy for Colon Cancer Diagnosis. Applied Sciences-Basel. 2020;10(16). doi: 10.3390/app10165401.
16. Rasmussen L, Wilhelmsen M, Christensen IJ, Andersen J, Jorgensen LN, Rasmussen M, et al. Protocol Outlines for Parts 1 and 2 of the Prospective Endoscopy III Study for the Early Detection of Colorectal Cancer: Validation of a Concept Based on Blood Biomarkers. JMIR Res Protoc. 2016;5(3):e182. doi: 10.2196/resprot.6346

17. Raza A, Khan AQ, Inchakalody VP, Mestiri S, Yoosuf Z, Bedhiafi T, et al. Dynamic liquid biopsy components as predictive and prognostic biomarkers in colorectal cancer. J Exp Clin Cancer Res. 2022;41(1):99. doi: 10.1186/s13046-022-02318-0.

18. Savva KV, Das B, Antonowicz S, Hanna GB, Peters CJ. Progress with Metabolomic Blood Tests for Gastrointestinal Cancer Diagnosis-An Assessment of Biomarker Translation. Cancer Epidemiol Biomarkers Prev. 2022;31(12):2095-105. doi: 10.1158/1055-9965.EPI-22-0307.

19. Suzuki M, Nishiumi S, Matsubara A, Azuma T, Yoshida M. Metabolome analysis for discovering biomarkers of gastroenterological cancer. J Chromatogr B Analyt Technol Biomed Life Sci. 2014;966:59-69. doi: 10.1016/j.jchromb.2014.02.042.

20. Ullah I, Yang L, Yin FT, Sun Y, Li XH, Li J, et al. Multi-Omics Approaches in Colorectal Cancer Screening and Diagnosis, Recent Updates and Future Perspectives. Cancers (Basel). 2022;14(22). doi: 10.3390/cancers14225545.

21. Wang H, Tso VK, Slupsky CM, Fedorak RN. Metabolomics and detection of colorectal cancer in humans: a systematic review. Future Oncol. 2010;6(9):1395-406. doi: 10.2217/fon.10.107.

22. Wang M, Long Z, Xue W, Peng C, Jiang T, Tian J, et al. Discovery of plasma biomarkers for colorectal cancer diagnosis via untargeted and targeted quantitative metabolomics. Clin Transl Med. 2022;12(4):e805. doi: 10.1002/ctm2.805.

23. Yu J, Zhao J, Zhang M, Guo J, Liu X, Liu L. Metabolomics studies in gastrointestinal cancer: a systematic review. Expert Rev Gastroenterol Hepatol. 2020;14(1):9-25. doi: 10.1080/17474124.2020.1700112.

24. Zhang A, Sun H, Yan G, Wang P, Han Y, Wang X. Metabolomics in diagnosis and biomarker discovery of colorectal cancer. Cancer Lett. 2014;345(1):17-20. doi: 10.1016/j.canlet.2013.11.011.

25. Zhang F, Zhang Y, Zhao W, Deng K, Wang Z, Yang C, et al. Metabolomics for biomarker discovery in the diagnosis, prognosis, survival and recurrence of colorectal cancer: a systematic review. Oncotarget. 2017;8(21):35460-72. doi: 10.18632/oncotarget.16727.

26. Zheng X, Xie G, Jia W. Metabolomic profiling in colorectal cancer: opportunities for personalized medicine. Per Med. 2013;10(7):741-55. doi: 10.2217/pme.13.73

27. Zhou L, Jiang Z, Zhang Z, Xing J, Wang D, Tang D. Progress of gut microbiome and its metabolomics in early screening of colorectal cancer. Clin Transl Oncol. 2023. doi: 10.1007/s12094-023-03097-6.

(B) No measurement of metabolites in pre-diagnostic samples

1. Amir Hashim, N. A., Ab-Rahim, S., Wan Ngah, W. Z., Nathan, S., Ab Mutalib, N. S., Sagap, I., . . . Mazlan, M. (2021). Global metabolomics profiling of colorectal cancer in Malaysian patients. Bioimpacts, 11(1), 33-43. doi:10.34172/bi.2021.05

2. Asante, I., Pei, H., Zhou, E., Liu, S., Chui, D., Yoo, E., . . . Louie, S. G. (2019). Exploratory metabolomic study to identify blood-based biomarkers as a potential screen for colorectal cancer. Mol Omics, 15(1), 21-29. doi:10.1039/c8mo00158h

3. Bestard-Escalas, J., Reigada, R., Reyes, J., de la Torre, P., Liebisch, G., & Barcelo-Coblijn, G. (2021). Fatty Acid Unsaturation Degree of Plasma Exosomes in Colorectal Cancer Patients: A Promising Biomarker. Int J Mol Sci, 22(10). doi:10.3390/ijms22105060

4. Chan, E. C., Koh, P. K., Mal, M., Cheah, P. Y., Eu, K. W., Backshall, A., . . . Keun, H. C. (2009). Metabolic profiling of human colorectal cancer using high-resolution magic angle spinning nuclear magnetic resonance (HR-MAS NMR) spectroscopy and gas chromatography mass spectrometry (GC/MS). J Proteome Res, 8(1), 352-361. doi:10.1021/pr8006232

5. Chen, C., Deng, L., Wei, S., Nagana Gowda, G. A., Gu, H., Chiorean, E. G., . . . Raftery, D. (2015). Exploring Metabolic Profile Differences between Colorectal Polyp Patients and Controls Using Seemingly Unrelated Regression. J Proteome Res, 14(6), 2492-2499. doi:10.1021/acs.jproteome.5b00059

6. Chen, H., Zhang, J., Zhou, H., Zhu, Y., Liang, Y., Zhu, P., & Zhang, Q. (2022). UHPLC-HRMS-based serum lipisdomics reveals novel biomarkers to assist in the discrimination between colorectal adenoma and cancer. Front Oncol, 12, 934145. doi:10.3389/fonc.2022.934145

7. Chen, H., Zhou, H., Liang, Y., Huang, Z., Yang, S., Wang, X., . . . Zhang, Q. (2023). UHPLC-HRMS-based serum untargeted lipidomics: Phosphatidylcholines and sphingomyelins are the main disturbed lipid markers to distinguish colorectal advanced adenoma from cancer. J Pharm Biomed Anal, 234, 115582. doi:10.1016/j.jpba.2023.115582
8. Chen, J. L., Fan, J., Yan, L. S., Guo, H. Q., Xiong, J. J., Ren, Y., & Hu, J. D. (2012). Urine Metabolite Profiling of Human Colorectal Cancer by Capillary Electrophoresis Mass Spectrometry Based on MRB. Gastroenterol Res Pract, 2012, 125890. doi:10.1155/2012/125890
9. Coker, O. O., Liu, C., Wu, W. K. K., Wong, S. H., Jia, W., Sung, J. J. Y., & Yu, J. (2022). Altered gut metabolites and microbiota interactions are implicated in colorectal carcinogenesis and can be non-invasive diagnostic biomarkers. Microbiome, 10(1), 35. doi:10.1186/s40168-021-01208-5
10. Crotti, S., Agnoletto, E., Cancemi, G., Di Marco, V., Traldi, P., Pucciarelli, S., . . . Agostini, M. (2016). Altered plasma levels of decanoic acid in colorectal cancer as a new diagnostic biomarker. Anal Bioanal Chem, 408(23), 6321-6328. doi:10.1007/s00216-016-9743-1
11. Cubiella, J., Clos-Garcia, M., Alonso, C., Martinez-Arranz, I., Perez-Cormenzana, M., Barrenetxea, Z., . . . Falcon-Perez, J. M. (2018). Targeted UPLC-MS Metabolic Analysis of Human Faeces Reveals Novel Low-Invasive Candidate Markers for Colorectal Cancer. Cancers (Basel), 10(9). doi:10.3390/cancers10090300
12. Del Boccio, P., Perrotti, F., Rossi, C., Cicalini, I., Di Santo, S., Zucchelli, M., . . . Pieragostino, D. (2017). Serum lipidomic study reveals potential early biomarkers for predicting response to chemoradiation therapy in advanced rectal cancer: A pilot study. Adv Radiat Oncol, 2(2), 118-124. doi:10.1016/j.adro.2016.12.005
13. Deng, L., Gu, H., Zhu, J., Nagana Gowda, G. A., Djukovic, D., Chiorean, E. G., & Raftery, D. (2016). Combining NMR and LC/MS Using Backward Variable Elimination: Metabolomics Analysis of Colorectal Cancer, Polyps, and Healthy Controls. Anal Chem, 88(16), 7975-7983. doi:10.1021/acs.analchem.6b00885
14. Deng, L., Ismond, K., Liu, Z., Constable, J., Wang, H., Alatise, O. I., . . . Chang, D. (2019). Urinary Metabolomics to Identify a Unique Biomarker Panel for Detecting Colorectal Cancer: A Multicenter Study. Cancer Epidemiol Biomarkers Prev, 28(8), 1283-1291. doi:10.1158/1055-9965.EPI-18-1291
15. Di Cesare, F., Vignoli, A., Luchinat, C., Tenori, L., & Saccenti, E. (2023). Exploration of Blood Metabolite Signatures of Colorectal Cancer and Polyposis through Integrated Statistical and Network Analysis. Metabolites, 13(2). doi:10.3390/metabo13020296
16. Di Giovanni, N., Meuwis, M. A., Louis, E., & Focant, J. F. (2020). Specificity of metabolic colorectal cancer biomarkers in serum through effect size. Metabolomics, 16(8), 88. doi:10.1007/s11306-020-01707-w
17. Di Giovanni, N., Meuwis, M. A., Louis, E., & Focant, J. F. (2023). Correlations for untargeted GC x GC-HRTOF-MS metabolomics of colorectal cancer. Metabolomics, 19(10), 85. doi:10.1007/s11306-023-02047-1
18. Feng, J., Gong, Z., Sun, Z., Li, J., Xu, N., Thorne, R. F., . . . Liu, G. (2023). Microbiome and metabolic features of tissues and feces reveal diagnostic biomarkers for colorectal cancer. Front Microbiol, 14, 1034325. doi:10.3389/fmicb.2023.1034325
19. Fernandes Messias, M. C., Mecatti, G. C., Figueiredo Angolini, C. F., Eberlin, M. N., Credidio, L., Real Martinez, C. A., . . . de Oliveira Carvalho, P. (2017). Plasma Lipidomic Signature of Rectal Adenocarcinoma Reveals Potential Biomarkers. Front Oncol, 7, 325. doi:10.3389/fonc.2017.00325
20. Gao, P., Zhou, C., Zhao, L., Zhang, G., & Zhang, Y. (2016). Tissue amino acid profile could be used to differentiate advanced adenoma from colorectal cancer. J Pharm Biomed Anal, 118, 349-355. doi:10.1016/j.jpba.2015.11.007
21. Gao, R., Wu, C., Zhu, Y., Kong, C., Zhu, Y., Gao, Y., . . . Qin, H. (2022). Integrated Analysis of Colorectal Cancer Reveals Cross-Cohort Gut Microbial Signatures and Associated Serum Metabolites. Gastroenterology, 163(4), 1024-1037 e1029. doi:10.1053/j.gastro.2022.06.069
22. Genua, F., Mirkovic, B., Mullee, A., Levy, M., Gallagher, W. M., Vodicka, P., & Hughes, D. J. (2021). Association of circulating short chain fatty acid levels with colorectal adenomas and colorectal cancer. Clin Nutr ESPEN, 46, 297-304. doi:10.1016/j.clnesp.2021.09.740
23. Goedert, J. J., Sampson, J. N., Moore, S. C., Xiao, Q., Xiong, X., Hayes, R. B., . . . Sinha, R. (2014). Fecal metabolomics: assay performance and association with colorectal cancer. Carcinogenesis, 35(9), 2089-2096. doi:10.1093/carcin/bgu131

24. Gu, J., Xiao, Y., Shu, D., Liang, X., Hu, X., Xie, Y., . . . Li, H. (2019). Metabolomics Analysis in Serum from Patients with Colorectal Polyp and Colorectal Cancer by (1)H-NMR Spectrometry. Dis Markers, 2019, 3491852. doi:10.1155/2019/3491852
25. Guo, J., Pan, Y., Chen, J., Jin, P., Tang, S., Wang, H., . . . Sheng, J. (2023). Serum metabolite signatures in normal individuals and patients with colorectal adenoma or colorectal cancer using UPLC-MS/MS method. J Proteomics, 270, 104741. doi:10.1016/j.jprot.2022.104741
26. Hama, K., Fujiwara, Y., Hayama, T., Ozawa, T., Nozawa, K., Matsuda, K., . . . Yokoyama, K. (2021). Very long-chain fatty acids are accumulated in triacylglycerol and nonesterified forms in colorectal cancer tissues. Sci Rep, 11(1), 6163. doi:10.1038/s41598-021-85603-w
27. Hussain, A., Xie, L., Deng, G., & Kang, X. (2023). Common alterations in plasma free amino acid profiles and gut microbiota-derived tryptophan metabolites of five types of cancer patients. Amino Acids, 55(9), 1189-1200. doi:10.1007/s00726-023-03308-y
28. Ikeda, A., Nishiumi, S., Shinohara, M., Yoshie, T., Hatano, N., Okuno, T., . . . Yoshida, M. (2012). Serum metabolomics as a novel diagnostic approach for gastrointestinal cancer. Biomed Chromatogr, 26(5), 548-558. doi:10.1002/bmc.1671
29. Jing, Y., Wu, X., Gao, P., Fang, Z., Wu, J., Wang, Q., . . . Cao, Y. (2017). Rapid differentiating colorectal cancer and colorectal polyp using dried blood spot mass spectrometry metabolomic approach. IUBMB Life, 69(5), 347-354. doi:10.1002/iub.1617
30. Khattab, R. H., Abo-Hammam, R. H., Salah, M., Hanora, A. M., Shabayek, S., & Zakeer, S. (2023). Multi-omics analysis of fecal samples in colorectal cancer Egyptians patients: a pilot study. BMC Microbiol, 23(1), 238. doi:10.1186/s12866-023-02991-x
31. Kim, E. R., Kwon, H. N., Nam, H., Kim, J. J., Park, S., & Kim, Y. H. (2019). Urine-NMR metabolomics for screening of advanced colorectal adenoma and early stage colorectal cancer. Sci Rep, 9(1), 4786. doi:10.1038/s41598-019-41216-y
32. Kondo, Y., Nishiumi, S., Shinohara, M., Hatano, N., Ikeda, A., Yoshie, T., . . . Yoshida, M. (2011). Serum fatty acid profiling of colorectal cancer by gas chromatography/mass spectrometry. Biomark Med, 5(4), 451-460. doi:10.2217/bmm.11.41
33. Kong, C., Liang, L., Liu, G., Du, L., Yang, Y., Liu, J., . . . Ma, Y. (2023). Integrated metagenomic and metabolomic analysis reveals distinct gut-microbiome-derived phenotypes in early-onset colorectal cancer. Gut, 72(6), 1129-1142. doi:10.1136/gutjnl-2022-327156
34. Krishnan, S. T., Winkler, D., Creek, D., Anderson, D., Kirana, C., Maddern, G. J., . . . Voelcker, N. H. (2023). Staging of colorectal cancer using lipid biomarkers and machine learning. Metabolomics, 19(10), 84. doi:10.1007/s11306-023-02049-z
35. Le Gall, G., Guttula, K., Kellingray, L., Tett, A. J., Ten Hoopen, R., Kemsley, E. K., . . . Narbad, A. (2018). Metabolite quantification of faecal extracts from colorectal cancer patients and healthy controls. Oncotarget, 9(70), 33278-33289. doi:10.18632/oncotarget.26022
36. Leichtle, A. B., Nuoffer, J. M., Ceglarek, U., Kase, J., Conrad, T., Witzigmann, H., . . . Fiedler, G. M. (2012). Serum amino acid profiles and their alterations in colorectal cancer. Metabolomics, 8(4), 643-653. doi:10.1007/s11306-011-0357-5
37. Li, C., Li, K., Xu, X., Qi, W., Hu, X., & Jin, P. (2021). A pilot study for colorectal carcinoma screening by instant metabolomic profiles using conductive polymer spray ionization mass spectrometry. Biochim Biophys Acta Mol Basis Dis, 1867(11), 166210. doi:10.1016/j.bbadis.2021.166210
38. Li, F., Qin, X., Chen, H., Qiu, L., Guo, Y., Liu, H., . . . Li, Z. (2013). Lipid profiling for early diagnosis and progression of colorectal cancer using direct-infusion electrospray ionization Fourier transform ion cyclotron resonance mass spectrometry. Rapid Commun Mass Spectrom, 27(1), 24-34. doi:10.1002/rcm.6420
39. Liesenfeld, D. B., Habermann, N., Toth, R., Owen, R. W., Frei, E., Staffa, J., . . . Ulrich, C. M. (2015). Changes in urinary metabolic profiles of colorectal cancer patients enrolled in a prospective cohort study (ColoCare). Metabolomics, 11(4), 998-1012. doi:10.1007/s11306-014-0758-3
40. Lin, L., Zeng, X., Liang, S., Wang, Y., Dai, X., Sun, Y., & Wu, Z. (2022). Biomarkers of coordinate metabolic reprogramming and the construction of a co-expression network in colorectal cancer. Ann Transl Med, 10(20), 1115. doi:10.21037/atm-22-4767
41. Lin, Y., Ma, C., Liu, C., Wang, Z., Yang, J., Liu, X., . . . Wu, R. (2016). NMR-based fecal metabolomics fingerprinting as predictors of earlier diagnosis in patients with colorectal cancer. Oncotarget, 7(20), 29454-29464. doi:10.18632/oncotarget.8762

42.	Liu, T., Peng, F., Yu, J., Tan, Z., Rao, T., Chen, Y., . . . Peng, J. (2019). LC-MS-based lipid profile in colorectal cancer patients: TAGs are the main disturbed lipid markers of colorectal cancer progression. Anal Bioanal Chem, 411(20), 5079-5088. doi:10.1007/s00216-019-01872-5
43.	Liu, T., Tan, Z., Yu, J., Peng, F., Guo, J., Meng, W., . . . Peng, J. (2020). A conjunctive lipidomic approach reveals plasma ethanolamine plasmalogens and fatty acids as early diagnostic biomarkers for colorectal cancer patients. Expert Rev Proteomics, 17(3), 233-242. doi:10.1080/14789450.2020.1757443
44.	Long, Y., Sanchez-Espiridion, B., Lin, M., White, L., Mishra, L., Raju, G. S., . . . Wu, X. (2017). Global and targeted serum metabolic profiling of colorectal cancer progression. Cancer, 123(20), 4066-4074. doi:10.1002/cncr.30829
45.	Ludwig, C., Ward, D. G., Martin, A., Viant, M. R., Ismail, T., Johnson, P. J., . . . Gunther, U. L. (2009). Fast targeted multidimensional NMR metabolomics of colorectal cancer. Magn Reson Chem, 47 Suppl 1, S68-73. doi:10.1002/mrc.2519
46.	Ma, Y. L., Qin, H. L., Liu, W. J., Peng, J. Y., Huang, L., Zhao, X. P., & Cheng, Y. Y. (2009). Ultra-high performance liquid chromatography-mass spectrometry for the metabolomic analysis of urine in colorectal cancer. Dig Dis Sci, 54(12), 2655-2662. doi:10.1007/s10620-008-0665-4
47.	Mirnezami, R., Jimenez, B., Li, J. V., Kinross, J. M., Veselkov, K., Goldin, R. D., . . . Darzi, A. (2014). Rapid diagnosis and staging of colorectal cancer via high-resolution magic angle spinning nuclear magnetic resonance (HR-MAS NMR) spectroscopy of intact tissue biopsies. Ann Surg, 259(6), 1138-1149. doi:10.1097/SLA.0b013e31829d5c45
48.	Miyagi, Y., Higashiyama, M., Gochi, A., Akaike, M., Ishikawa, T., Miura, T., . . . Okamoto, N. (2011). Plasma free amino acid profiling of five types of cancer patients and its application for early detection. Plos One, 6(9), e24143. doi:10.1371/journal.pone.0024143
49.	Nannini, G., Meoni, G., Tenori, L., Ringressi, M. N., Taddei, A., Niccolai, E., . . . Amedei, A. (2021). Fecal metabolomic profiles: A comparative study of patients with colorectal cancer vs adenomatous polyps. World J Gastroenterol, 27(38), 6430-6441. doi:10.3748/wjg.v27.i38.6430
50.	Ning, W., Li, H., Meng, F., Cheng, J., Song, X., Zhang, G., . . . Dong, F. (2017). Identification of differential metabolic characteristics between tumor and normal tissue from colorectal cancer patients by gas chromatography-mass spectrometry. Biomed Chromatogr, 31(11). doi:10.1002/bmc.3999
51.	Nishiumi, S., Kobayashi, T., Ikeda, A., Yoshie, T., Kibi, M., Izumi, Y., . . . Yoshida, M. (2012). A novel serum metabolomics-based diagnostic approach for colorectal cancer. Plos One, 7(7), e40459. doi:10.1371/journal.pone.0040459
52.	Phua, L. C., Chue, X. P., Koh, P. K., Cheah, P. Y., Ho, H. K., & Chan, E. C. (2014). Non-invasive fecal metabonomic detection of colorectal cancer. Cancer Biol Ther, 15(4), 389-397. doi:10.4161/cbt.27625
53.	Rachieriu, C., Eniu, D. T., Mois, E., Graur, F., Socaciu, C., Socaciu, M. A., & Hajjar, N. A. (2021). Lipidomic Signatures for Colorectal Cancer Diagnosis and Progression Using UPLC-QTOF-ESI(+)MS. Biomolecules, 11(3). doi:10.3390/biom11030417
54.	Ritchie, S. A., Ahiahonu, P. W., Jayasinghe, D., Heath, D., Liu, J., Lu, Y., . . . Goodenowe, D. B. (2010). Reduced levels of hydroxylated, polyunsaturated ultra long-chain fatty acids in the serum of colorectal cancer patients: implications for early screening and detection. BMC Med, 8, 13. doi:10.1186/1741-7015-8-13
55.	Song, E. M., Byeon, J. S., Lee, S. M., Yoo, H. J., Kim, S. J., Lee, S. H., . . . Jeong, J. Y. (2018). Fecal Fatty Acid Profiling as a Potential New Screening Biomarker in Patients with Colorectal Cancer. Dig Dis Sci, 63(5), 1229-1236. doi:10.1007/s10620-018-4982-y
56.	Tan, B., Qiu, Y., Zou, X., Chen, T., Xie, G., Cheng, Y., . . . Jia, W. (2013). Metabonomics identifies serum metabolite markers of colorectal cancer. J Proteome Res, 12(6), 3000-3009. doi:10.1021/pr400337b
57.	Uchiyama, K., Naito, Y., Yagi, N., Mizushima, K., Higashimura, Y., Hirai, Y., . . . Itoh, Y. (2021). Identification of colorectal neoplasia by using serum bile acid profile. Biomarkers, 26(5), 462-467. doi:10.1080/1354750X.2021.1917663
58.	Uchiyama, K., Yagi, N., Mizushima, K., Higashimura, Y., Hirai, Y., Okayama, T., . . . Naito, Y. (2017). Serum metabolomics analysis for early detection of colorectal cancer. J Gastroenterol, 52(6), 677-694. doi:10.1007/s00535-016-1261-6
59.	Udo, R., Katsumata, K., Kuwabara, H., Enomoto, M., Ishizaki, T., Sunamura, M., . . . Tsuchida, A. (2020). Urinary charged metabolite profiling of colorectal cancer using capillary electrophoresis-mass spectrometry. Sci Rep, 10(1), 21057. doi:10.1038/s41598-020-78038-2

60. Wang, Z., Lin, Y., Liang, J., Huang, Y., Ma, C., Liu, X., & Yang, J. (2017). NMR-based metabolomic techniques identify potential urinary biomarkers for early colorectal cancer detection. Oncotarget, 8(62), 105819-105831. doi:10.18632/oncotarget.22402
61. Wu, J., Wu, M., & Wu, Q. (2020). Identification of potential metabolite markers for colon cancer and rectal cancer using serum metabolomics. J Clin Lab Anal, 34(8), e23333. doi:10.1002/jcla.23333
62. Yachida, S., Mizutani, S., Shiroma, H., Shiba, S., Nakajima, T., Sakamoto, T., . . . Yamada, T. (2019). Metagenomic and metabolomic analyses reveal distinct stage-specific phenotypes of the gut microbiota in colorectal cancer. Nat Med, 25(6), 968-976. doi:10.1038/s41591-019-0458-7
63. Yang, C., Zhou, S., Zhu, J., Sheng, H., Mao, W., Fu, Z., & Chen, Z. (2022). Plasma lipid-based machine learning models provides a potential diagnostic tool for colorectal cancer patients. Clin Chim Acta, 536, 191-199. doi:10.1016/j.cca.2022.09.002
64. Yang, Y., Wang, Z., Li, X., Lv, J., Zhong, R., Gao, S., . . . Chen, W. (2023). Profiling the metabolic disorder and detection of colorectal cancer based on targeted amino acids metabolomics. J Transl Med, 21(1), 824. doi:10.1186/s12967-023-04604-7
65. Yuan, F., Kim, S., Yin, X., Zhang, X., & Kato, I. (2020). Integrating Two-Dimensional Gas and Liquid Chromatography-Mass Spectrometry for Untargeted Colorectal Cancer Metabolomics: A Proof-of-Principle Study. Metabolites, 10(9). doi:10.3390/metabo10090343
66. Yuan, W., Chen, Y., Zhuang, D., Zeng, H., Lin, X., Hong, S., . . . Lin, F. (2023). UHPLC-MS/MS-based central carbon metabolism unveils the biomarkers related to colon cancer. Cell Mol Biol (Noisy-le-grand), 69(9), 167-171. doi:10.14715/cmb/2023.69.9.25
67. Zamani, Z., Arjmand, M., Vahabi, F., Eshaq Hosseini, S. M., Fazeli, S. M., Iravani, A., . . . Sadeghi, S. (2014). A metabolic study on colon cancer using (1)h nuclear magnetic resonance spectroscopy. Biochem Res Int, 2014, 348712. doi:10.1155/2014/348712
68. Zhang, C., Zhou, S., Chang, H., Zhuang, F., Shi, Y., Chang, L., . . . Hong, T. (2021). Metabolomic Profiling Identified Serum Metabolite Biomarkers and Related Metabolic Pathways of Colorectal Cancer. Dis Markers, 2021, 6858809. doi:10.1155/2021/6858809
69. Zhang, L., Feng, Z., Li, Y., Lv, C., Li, C., Hu, Y., . . . Song, L. (2023). Salivary and fecal microbiota: potential new biomarkers for early screening of colorectal polyps. Front Microbiol, 14, 1182346. doi:10.3389/fmicb.2023.1182346
70. Zhang, L., Liu, J., Deng, M., Chen, X., Jiang, L., Zhang, J., . . . Qiu, Y. (2023). Enterococcus faecalis promotes the progression of colorectal cancer via its metabolite: biliverdin. J Transl Med, 21(1), 72. doi:10.1186/s12967-023-03929-7
71. Zhang, L., Zheng, J., Ismond, K. P., MacKay, S., LeVatte, M., Constable, J., . . . Wishart, D. S. (2023). Identification of urinary biomarkers of colorectal cancer: Towards the development of a colorectal screening test in limited resource settings. Cancer Biomark, 36(1), 17-30. doi:10.3233/CBM-220034
72. Zhang, L. J., Chen, B., Zhang, J. J., Li, J., Yang, Q., Zhong, Q. S., . . . Cai, C. (2017). Serum polyunsaturated fatty acid metabolites as useful tool for screening potential biomarker of colorectal cancer. Prostaglandins Leukot Essent Fatty Acids, 120, 25-31. doi:10.1016/j.plefa.2017.04.003
73. Zhang, Y., Du, Y., Song, Z., Liu, S., Li, W., Wang, D., & Suo, J. (2020). Profiling of serum metabolites in advanced colon cancer using liquid chromatography-mass spectrometry. Oncol Lett, 19(6), 4002-4010. doi:10.3892/ol.2020.11510
74. Zhang, Y., He, C., Qiu, L., Wang, Y., Qin, X., Liu, Y., & Li, Z. (2016). Serum Unsaturated Free Fatty Acids: A Potential Biomarker Panel for Early-Stage Detection of Colorectal Cancer. J Cancer, 7(4), 477-483. doi:10.7150/jca.13870
75. Zhao, Z., Bai, J., Liu, C., Wang, Y., Wang, S., Zhao, F., & Gu, Q. (2023). Metabolomics analysis of amino acid and fatty acids in colorectal cancer patients based on tandem mass spectrometry. J Clin Biochem Nutr, 73(2), 161-171. doi:10.3164/jcbn.22-110
76. Zhou, H., Nong, Y., Zhu, Y., Liang, Y., Zhang, J., Chen, H., . . . Zhang, Q. (2022). Serum untargeted lipidomics by UHPLC-ESI-HRMS aids the biomarker discovery of colorectal adenoma. BMC Cancer, 22(1), 314. doi:10.1186/s12885-022-09427-1
77. Zhu, G., Wang, Y., Wang, W., Shang, F., Pei, B., Zhao, Y., . . . Fan, Z. (2021). Untargeted GC-MS-Based Metabolomics for Early Detection of Colorectal Cancer. Front Oncol, 11, 729512. doi:10.3389/fonc.2021.729512

78. Zhu, Y., Wang, L., Nong, Y., Liang, Y., Huang, Z., Zhu, P., & Zhang, Q. (2021). Serum Untargeted UHPLC-HRMS-Based Lipidomics to Discover the Potential Biomarker of Colorectal Advanced Adenoma. Cancer Manag Res, 13, 8865-8878. doi:10.2147/CMAR.S336322
79. Zhu, Y. F., Zhou, H. L., Chen, H. W., Zhang, J. H., Liang, Y. X., Yang, S. Y., . . . Zhang, Q. S. (2023). Global serum metabolomic and lipidomic analyses reveal lipid perturbations and potential biomarkers of the colorectal cancer by adenoma-carcinoma sequence. Chinese Journal of Analytical Chemistry, 51(7), 100270. doi:10.1016/j.cjac.2023.100270
**(C) No blood, urine, or stool samples**
1. Batty C, Cauchi M, Lourenço C, Hunter J, Turner C. Use of the Analysis of the Volatile Faecal Metabolome in Screening for Colorectal Cancer. Plos One. 2015;10(6):e0130301. doi: 10.1371/journal.pone.0130301.
2. Bond A, Greenwood R, Lewis S, Corfe B, Sarkar S, O'Toole P, et al. Volatile organic compounds emitted from faeces as a biomarker for colorectal cancer. Aliment Pharmacol Ther. 2019;49(8):1005-12. doi: 10.1111/apt.15140.
3. Boulind CE, Gould O, de Lacy Costello B, Allison J, White P, Ewings P, et al. Urinary Volatile Organic Compound Testing in Fast-Track Patients with Suspected Colorectal Cancer. Cancers (Basel). 2022;14(9). doi: 10.3390/cancers14092127.
4. Clos-Garcia M, Garcia K, Alonso C, Iruarrizaga-Lejarreta M, D'Amato M, Crespo A, et al. Integrative Analysis of Fecal Metagenomics and Metabolomics in Colorectal Cancer. Cancers (Basel). 2020;12(5). doi: 10.3390/cancers12051142
5. Boulind CE, Gould O, de Lacy Costello B, Allison J, White P, Ewings P, et al. Urinary Volatile Organic Compound Testing in Fast-Track Patients with Suspected Colorectal Cancer. Cancers (Basel). 2022;14(9). doi: 10.3390/cancers14092127.
**(D) Lack of sufficient statistical data**
1. Erben V, Poschet G, Schrotz-King P, Brenner H. Comparing Metabolomics Profiles in Various Types of Liquid Biopsies among Screening Participants with and without Advanced Colorectal Neoplasms. Diagnostics (Basel). 2021;11(3). doi: 10.3390/diagnostics11030561.
2. Perttula K, Edmands WM, Grigoryan H, Cai X, Iavarone AT, Gunter MJ, et al. Evaluating Ultra-long-Chain Fatty Acids as Biomarkers of Colorectal Cancer Risk. Cancer Epidemiol Biomarkers Prev. 2016;25(8):1216-23. doi: 10.1158/1055-9965.EPI-16-0204.
3. Perttula K, Schiffman C, Edmands WMB, Petrick L, Grigoryan H, Cai X, et al. Untargeted lipidomic features associated with colorectal cancer in a prospective cohort. BMC Cancer. 2018;18(1):996. doi: 10.1186/s12885-018-4894-4.

Supplementary Table 4. QUADAS-2 Risk of Bias assessment

	Risk of bias				Applicability concerns		
**First author, Year ref.**	Patient selection	Index test	Reference standard	Flow and timing	Patient selection	Index test	Reference standard
Cai, 2006 [14]	☺	?	?	☺	☺	☺	☺
Cross, 2014 [15]	☺	?	?	☺	☺	☺	☺
Kühn, 2016 [17]	☺	☺	?	☺	☺	☺	☺
Myte, 2017 [20]	?	☺	☺	☹	☺	☺	☺
Pickens, 2017 [37]	☺	?	☺	?	☹	☺	☺
Geijsen, 2019 [16]	?	?	?	?	☹	☺	☺
Kühn, 2020 [28]	☺	☺	☺	?	☺	☺	☺
McCullough, 2021 [19]	☺	☺	☺	?	☺	☺	☺
Papadimitriou, 2021 [29]	?	?	☺	☹	☹	☺	☺
Tevini, 2022 [39]	?	?	☺	?	☺	☺	☺
Hang, 2022 [36]	?	?	?	☹	☹	☺	☺
Pham, 2022 [21]	☺	☺	☺	?	☹	☺	☺
Vidman, 2023 [26]	☺	☺	☺	☹	☺	☺	☹
Eisner, 2013 [34]	?	?	☺	☹	☹	☺	☺
Wang, 2014 [35]	☺	☺	☺	☹	☺	☺	☺
Amiot, 2015 [38]	☺	?	?	☺	☺	☺	☺
Farshidfar, 2016 [30]	☺	☺	☺	☺	☹	☺	☺
Deng, 2017a [32]	☺	?	?	☺	☺	☺	☺
Deng, 2017b [33]	☺	?	?	☹	☹	☺	☺
Troisi, 2022 [25]	☹	☹	☺	?	☺	☺	☺
Rothwell, 2022 [22]	☺	☺	☺	?	☹	☺	☺
Telleria, 2022 [24]	?	?	☺	☺	☺	☺	☺
Liu, 2023 [31]	?	?	?	?	☺	☺	☺
Xie, 2023 [27]	☹	?	☺	?	☹	☺	?
Shu, 2018 [23]	☺	☺	☺	☹	☺	☺	☺
Loftfield, 2022 [18]	☺	?	?	☺	☺	☺	☺

☺ Low concern/risk

? Unclear concern/risk

☹ High concern/risk

Supplementary Table 5. Covariates included in the panel of metabolites/in the analysis for the individual metabolites

**First author, Year ref.**	Age	Sex	Smoking	Alcohol consumption	Weight-related covariates	Diet-related covariates	Educational level	Physical activity	Fasting time before sample collection	Anti-inflammatory medication	Others
**Individual Metabolites**											
Cai, 2006 [14]	-	*	-	-	-	-	-	-	-	-	
Cross, 2014 [15]	x	-	-	-	x	-	-	-	-	-	
Kühn, 2016 [17]	x	x	x	x	x	x	x	-	-	x	
Myte, 2017 [20]	-	-	x	x	x	-	-	x	-	-	
Pickens, 2017 [37]	x	*	x	-	-	-	-	-	-	-	
Geijsen, 2019 [16]	x	x	x	-	x	-	-	-	-	-	
Kühn, 2020 [28]	-	-	x	-	x	x	x	x	-	-	
McCullough, 2021 [19]	-	-	x	x	x	x	-	x	x	x	
Papadimitriou, 2021 [29]	x	x	x	-	x	x	x	-	-	-	
Tevini, 2022 [39]	-	-	-	-	-	-	-	-	-	-	
Hang, 2022 [36]	x	*	-	-	-	-	-	-	-	-	CRC screening
Pham, 2022 [21]	-	-	-	-	-	-	-	-	-	-	
Vidman, 2023 [26]	-	-	x	x	x	-	x	x	-	-	Diabetes
**Metabolite Panel**											
Eisner, 2013 [34]	-	x	x	-	-	-	-	-	-	-	Gastro-intestinal bleeding
Wang, 2014 [35]	x	-	-	-	-	-	-	-	-	-	
Amiot, 2015 [38]	-	-	-	-	-	-	-	-	-	-	
Farshidfar, 2016 [30]	x	-	-	-	-	-	-	-	-	-	
Deng, 2017a [32]	x	x	x	-	-	-	-	-	-	-	
Deng, 2017b [33]	x	x	x	-	-	-	-	-	-	-	
Troisi, 2022 [25]	-	-	-	-	-	-	-	-	-	-	
Rothwell, 2022 [22]	-	-	x	x	x	x	x	-	-	-	Height
Telleria, 2022 [24]	-	-	-	-	-	-	-	-	-	-	Hemoglobin
Liu, 2023 [31]	-	-	-	-	-	-	-	-	-	-	
Xie, 2023 [27]	-	-	-	-	-	-	-	-	-	-	
**Individual Metabolites & Metabolite Panel**											
Shu, 2018 [23]	x	-	x	x	x	x	-	x	x	-	
Loftfield, 2022 [18]	x	-	x	x	x	x	x	x	-	-	

Notes. * only included male/female participants; Abbreviations. CRC, colorectal cancer
